# Synthesis of 2*H*-furo[2,3-*c*]pyrazole ring systems through silver(I) ion-mediated ring-closure reaction

**DOI:** 10.3762/bjoc.15.62

**Published:** 2019-03-14

**Authors:** Vaida Milišiūnaitė, Rūta Paulavičiūtė, Eglė Arbačiauskienė, Vytas Martynaitis, Wolfgang Holzer, Algirdas Šačkus

**Affiliations:** 1 Department of Organic Chemistry, Kaunas University of Technology, Radvilėnų pl. 19, Kaunas LT-50254, Lithuania; 2 Institute of Synthetic Chemistry, Kaunas University of Technology, K. Baršausko g. 59, Kaunas LT-51423, Lithuania; 3 Department of Pharmaceutical Chemistry, University of Vienna, Althanstrasse 14, Vienna 1090, Austria

**Keywords:** 5-*endo-dig* cyclization, 2*H*-furo[2,3-*c*]pyrazole, pyrazole, silver(I) catalyst, Sonogashira coupling

## Abstract

Fused pyrazole ring systems are common structural motifs of numerous pharmaceutically important compounds. Nevertheless, access to derivatives of the aromatic 2*H*-furo[2,3-*c*]pyrazole ring system is still quite limited, and their chemistry and functional properties remain largely underexplored. The current study investigates routes to construct this system from easily accessible starting materials using metal-catalyzed reactions. A simple and efficient procedure to access the 2*H*-furo[2,3-*c*]pyrazole ring system was developed by employing the silver(I) ion-mediated ring-closure reaction of 4-alkynyl-3-hydroxy-1-phenyl-1*H*-pyrazoles as a key step. The required intermediate hydroxyalkynyl substrates for this reaction were prepared by a Pd-catalyzed coupling of 4-iodo-1-phenyl-1*H*-pyrazol-3-ol with ethyne derivatives. The structures of the obtained target compounds were unequivocally confirmed by detailed ^1^H, ^13^C and ^15^N NMR spectroscopic experiments, HRMS and a single-crystal X-ray diffraction analyses. This silver(I)-mediated 5-*endo-dig* cyclization of readily available 4-alkynyl-3-hydroxy-1*H*-pyrazoles can be used as an efficient method to access many novel 2,5-disubstituted 2*H*-furo[2,3-*c*]pyrazoles.

## Introduction

Heterocyclic ring systems possessing a pyrazole ring fused to an oxygen-containing six-membered heterocycle are present in a wide variety of biologically active compounds. For example, the 1,4- and 2,4-dihydropyrano[2,3-*c*]pyrazole ring systems [1] often represent the main structural motifs of anticancer [2–4], anti-inflammatory [5], and antidiabetic agents [6]. The numerous known methods for the preparation of these compounds are generally based on multicomponent reactions of an aromatic aldehyde, a β-keto ester, a hydrazine and malononitrile [7]. In a similar reaction using a pyridinium ylide instead of malononitrile, the dihydro-1*H*-furo[2,3-*c*]pyrazole system, consisting of an aromatic pyrazole ring fused with a five-membered 2,3-dihydrofuran ring, was formed [8]. Derivatives of the latter system are known for their antimicrobial [9] and antiproliferative activities [10]. Nevertheless, methods to access 2*H*-furo[2,3-*c*]pyrazoles, where both the fused pyrazole and furan moieties are aromatic, are still quite limited, and their chemistry and functional properties remain largely underexplored. Huang et al. have reported the synthesis and the evaluation of antiplatelet and anti-allergic activities of 2,3,4-trisubstituted 2*H*-furo[2,3-*c*]pyrazole-5-carboxylic acids and related carboxamides [11–12]. The aforementioned carboxylic acids have been prepared by bromination of the corresponding pyrano[2,3-*c*]pyrazol-6(1*H*)-one derivatives followed by heating of the obtained 5-bromo derivatives in the presence of sodium alkoxide [11]. Notably, the 2*H*-furo[2,3-*c*]pyrazole ring system is structurally similar to the benzo[*b*]furan system, which is a privileged motif in natural products and biologically active compounds [13–14]. As a result, numerous strategies have been developed for the construction of benzo[*b*]furan and its derivatives [15–18]. For example, Damera et al. reported an efficient synthesis of 2-substituted benzo[*b*]furans with good yields by the base-promoted cyclization of easily accessible 2-alkynylphenols [19]. Recently, 2-arylbenzo[*b*]furans were conveniently synthesized by the one-pot tandem Hiyama alkynylation/cyclization reaction between 2-iodophenol and (triethoxysilyl)alkynes [20]. In recent years silver and gold salts have found application as versatile and mild catalysts to access the benzo[*b*]furan ring system through intramolecular cyclization of 2-alkynylphenol substrates [21], including preparation of 2-benzofuranmethanamines [22] and 4-indolylbenzo[*b*]furans [23] in the presence of AgNO_3_ and AgOTf, respectively, and 2-phenylbenzo[*b*]furans, where AuCl_3_ or a mixture thereof with AgOTf has been used to promote the appropriate cyclization [24].

In the present work, we describe a method for the construction of the 2*H*-furo[2,3-*c*]pyrazole ring system by a Sonogashira-type alkynylation of 4-iodopyrazol-3-ol and subsequent intramolecular 5-*endo-dig* cyclization of the obtained hydroxyalkynyl substrate mediated by a Ag(I) catalyst.

## Results and Discussion

**Scheme 1:**
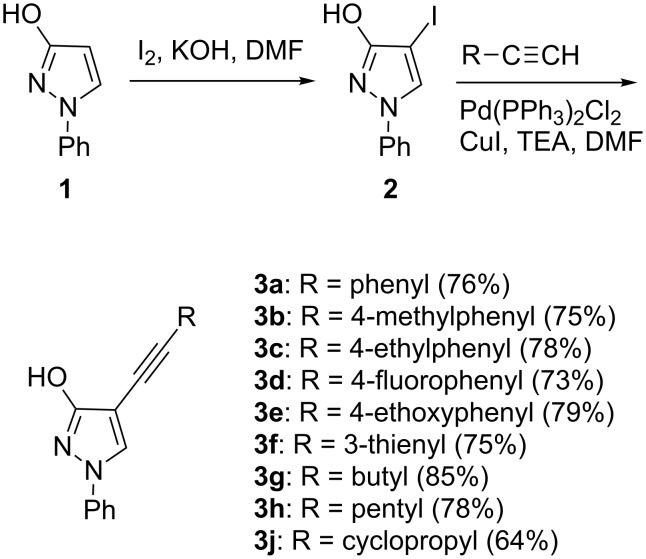
Preparation of hydroxyalkynyl substrates from 1-phenyl-1*H*-pyrazol-3-ol (**1**).

The synthetic strategy designed to construct the 2*H*-furo[2,3-*c*]pyrazole ring system employs a hydroxyethynyl substrate that contains adjacent ethyne and hydroxy groups on the pyrazole core (Scheme 1). As a starting material, we used 1-phenyl-1*H*-pyrazol-3-ol (**1**), which is readily accessible from the oxidation of 1-phenyl-3-pyrazolidinone [25–26]. Recently, we used this scaffold to obtain the 2*H-*pyrazolo[4,3-*c*]pyridine [27–28], pyrazolo[4,3-*f*][1,2,3]triazolo[5,1-*c*][1,4]oxazepine [29] and pyrazolo[4’,3’:3,4]pyrido[1,2-*a*]benzimidazole [30] ring systems as well as to prepare building blocks for the construction of optoelectronic materials and fluorescent organic nanoparticles [31–34].

The iodination of **1** with iodine in the presence of KOH in DMF afforded 4-iodo-1*H*-pyrazol-3-ol **2** [26]. We have previously shown that the Sonogashira-type coupling of the aforementioned iodinated compound with phenylacetylene under standard reaction conditions (Pd(PPh_3_)_2_Cl_2_, CuI, and TEA) gives 4-(phenylethynyl)-substituted pyrazolol **3a** [26]. When various het(aryl)- and alkylacetylenes were used in the coupling with **2** under the same reaction conditions, compounds **3b–j** were obtained in 64–85% yield (Scheme 1).

Having prepared the series of hydroxyethynyl substrates **3a–j**, the optimal conditions for the subsequent cyclization reaction were next investigated using **3a** as the model compound (Table 1). First, we attempted the desired cyclization of **3a** to 2*H*-furo[2,3-*c*]pyrazole **4a** using the synthetic protocol involving Cs_2_CO_3_ in dry DMF at 60 °C, which has been previously successfully employed for the preparation of benzo[*b*]furans by the cyclization of *o*-alkynylphenols [19]. Unfortunately, no addition of the hydroxy group across the carbon–carbon triple bond was observed even after heating the reaction mixture under analogous reaction conditions for 24 hours (Table 1, entry 1).

**Table 1:** Cyclization of compound **3a**.

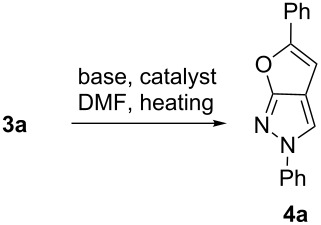					

Entry	Base	Catalyst	Temp (°C)	*t* (h)	Yield **4a**

1	Cs_2_CO_3_	–	60	24	no product
2	Cs_2_CO_3_	–	120	96	traces
3	K_2_CO_3_	–	120	96	47%
4	K_2_CO_3_	[(Ph_3_P)Au]Cl (10 mol %)	120	14	69%
5	K_2_CO_3_	AgOTf (10 mol %)	120	14	92%
6	K_2_CO_3_	AgOTf (10 mol %)	80	96	15%
7	–	AgOTf (1 equiv)	120	96	traces
8	Cs_2_CO_3_	AgOTf (10 mol %)	120	96	69%

The formation through a 5-*endo-dig* cyclization of some amount of compound **4a** was detected by LC–MS measurements only when the reaction mixture contained Cs_2_CO_3_ as a base and was heated at 120 °C for 4 days (Table 1, entry 2). Surprisingly, when K_2_CO_3_ was used instead of Cs_2_CO_3_, the desired 2-phenyl-2*H*-furo[2,3-*c*]pyrazole was obtained in a significantly higher yield (47%, Table 1, entry 3).

Some of the most effective catalysts for the electrophilic activation of alkynes under homogeneous conditions are gold(I) [35] and silver(I) [36] salts or complexes, and a broad range of versatile synthetic methods has been developed for the construction of carbon–heteroatom bonds using these types of catalysts. For example, the gold(I) catalyst [(Ph_3_PAu)_3_O]BF_4_ was applied in the regioselective intramolecular cyclization of alkynols to construct bicyclic ethers [37], while the silver(I) catalyst AgOTf efficiently catalyzed the intramolecular cyclization of phenoxyethynyl diols into 2,3-unsaturated lactones [38]. In our case, the addition of 10 mol % chloro(triphenylphosphine)gold(I) improved the yield of product **4a** to 69% (Table 1, entry 4). However the best results were obtained when AgOTf was used as a catalyst. In this case, the target product **4a** was obtained in an excellent 92% yield (Table 1, entry 5).

It is important to note that the reaction temperature had a significant effect on the yield of the product. When the temperature was lowered to 80 °C, the yield of the product did not exceeded 15% (Table 1, entry 6). The presence of a base also plays a crucial role in the cyclization described herein, and the transformation of **3a** to **4a** did not occur in the presence of only the catalyst and without base (Table 1, entry 7). Finally, the reaction optimization experiments showed that the Cs_2_CO_3_/AgOTf system does not offer any advantages for this cyclization compared to the K_2_CO_3_/AgOTf system (Table 1, entry 8).

**Scheme 2:**
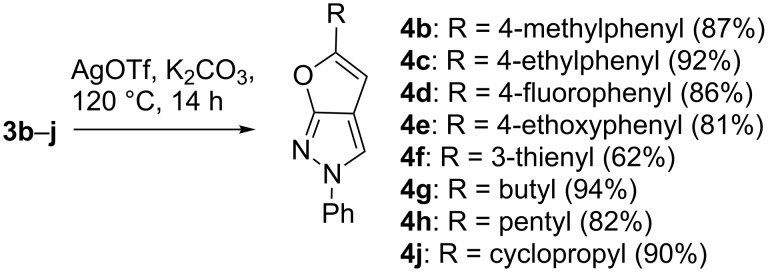
Cyclization of hydroxyalkynyl substrates to 2,5-disubstituted 2*H*-furo[2,3-*c*]pyrazoles.

With the optimized conditions for the 5-*endo-dig* cyclization reaction identified, the scope of this transformation for the preparation of several 2,5-disubstituted 2*H*-furo[2,3-*c*]pyrazoles was explored (Scheme 2). For substrates **3b**–**j**, the reactions were complete after 14 hours at 120 °C, and products **4b**–**j** were generated in fair to excellent yields.

The structural assignments of **4a**–**j** were based on multinuclear NMR and IR spectroscopy as well as high-resolution mass spectrometry (HRMS) data and a single-crystal X-ray diffraction analysis. The ^1^H NMR spectra of compounds **4a**–**j** revealed the characteristic H-3 proton singlet in the δ 7.60–7.77 ppm region as well as a H-4 proton singlet in the δ 6.06–6.74 ppm region. The ^13^C NMR spectra of **4a**–**j** exhibited signals for the five carbon atoms of the 2*H*-furo[2,3-*c*]pyrazole ring system in the regions of δ 115.0–116.3 for C-3, δ 113.1–113.9 for C-3a, δ 94.5–96.8 for C-4, δ 156.1–163.8 for C-5 and δ 168.4–169.0 ppm for C-6a. The ^15^N spectra of **4b**–**e** exhibited signals for two nitrogen atoms in the regions of δ −169.4 to −172.6 for N-2 and δ −127.5 to −128.0 ppm for N-1.

**Figure 1:**
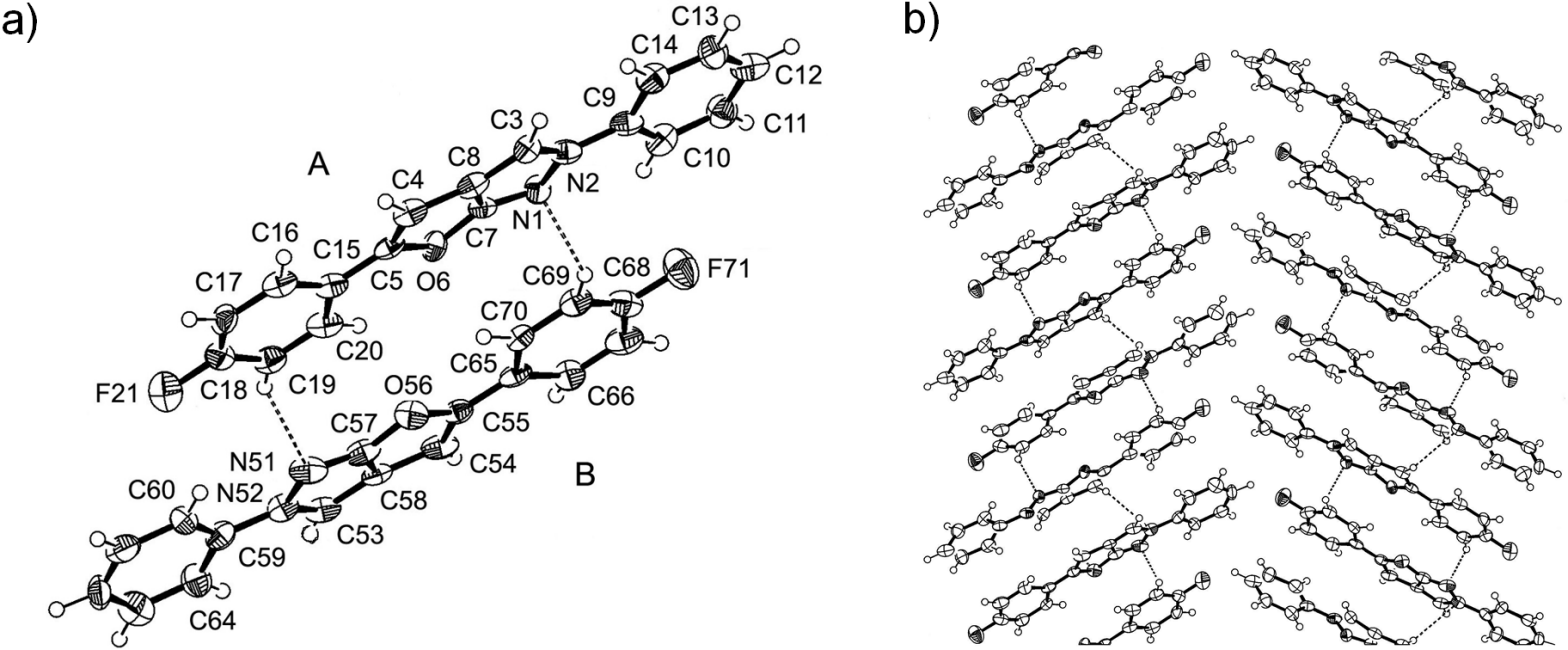
a) ORTEP diagram of the asymmetric unit consisting of two independent molecules **4d**(**A**) and **4d**(**B**); b) view (normal to (100)) of molecular packing in the crystal.

The structure of **4d** was investigated additionally by single crystal X-ray analysis (Figure 1) [39].

The asymmetric unit of the crystal consists of two molecules **4d**(**A**) and **4d**(**B**) held together by the weak CH···N type hydrogen bonds C19–H···N51 (C···N = 3.470(11) Å, H···N = 2.60 Å, C–H···N = 151°) and C69–H···N1 (C···N = 3.355(11) Å, H···N = 2.51 Å, C–H···N = 144°), respectively, whose length and angles are typical for crystals of aza-heterocycles [40]. Although at first glance the molecules **4d**(**A**) and **4d**(**B**) appear to be identical, they differ in bond lengths and dihedral angles Φ from each other. The corresponding bond lengths of 2*H*-furo[2,3-*c*]pyrazole systems in **4d**(**A**) and **4d**(**B**) are given in Table 2.

**Table 2:** Selected bond lengths of the 2*H*-furo[2,3-*c*]pyrazole core.

Molecule **4d**(**A**)	*d*, Å	Molecule **4d**(**B**)	*d*, Å

N1–N2	1.391(7)	N51–N52	1.407(8)
N2–C3	1.378(8)	N52–C53	1.358(9)
C3–C8	1.378(10)	C53–C58	1.371(11)
C8–C4	1.457(11)	C58–C54	1.450(11)
C4–C5	1.362(8)	C54–C55	1.337(9)
C5–O6	1.401(8)	C55–O56	1.424(8)
O6–C7	1.354(9)	O56–C57	1.365(10)
C7–N1	1.317(11)	C57–N51	1.298(12)
C7–C8	1.396(8)	C57–C58	1.405(9)

The lengths of the C–F bonds for molecules **4d**(**A**) and **4d**(**B**) are 1.356(10) Å and 1.392(11) Å, respectively. All ring atoms of the 2*H*-furo[2,3-*c*]pyrazole moiety lie in almost one plane, but the phenyl substituents are slightly turned in relation to this plane (Table 3).

**Table 3:** Dihedral angles Φ formed by the phenyls and the 2*H*-furo[2,3-*c*]pyrazole core in **4d**(**A**) and **4d**(**B**).

Substitutent	Molecule **4d**(**A**)	Φ, deg	Molecule **4d**(**B**)	Φ, deg

phenyl	C10–C9–N2–N1	7.64	C60–C59–N52–N51	6.97
	C14–C9–N2–C3	10.09	C64–C59–N52–C53	10.68
4-fluorophenyl	C16–C15–C5–C4	9.71	C66–C65–C55–C54	5.06
	C20–C15–C5–O6	6.51	C70–C65–C55–O56	8.89

The molecules in the crystal are located in columns made up of asymmetric units held by hydrogen bonds (Figure 1b).

## Conclusion

In conclusion, we have demonstrated a new, three-step synthetic route to 2*H*-furo[2,3-*c*]pyrazoles starting from commercially available 1-phenylpyrazol-3-ol. Iodination of the latter compound with iodine in DMF smoothly afforded 1-phenyl-4-iodopyrazol-3-ol, which can undergo a Pd-catalyzed coupling with terminal alkynes to give the corresponding 4-alkynyl-3-hydroxy-1-phenyl-1*H*-pyrazoles. The desired 5-*endo-dig* cyclization leading to the formation of the 2*H*-furo[2,3-*c*]pyrazole ring system is easily achieved by heating the aforementioned hydroxyalkynyl substrates with a base in DMF in the presence of a silver(I) catalyst.

## Supporting Information

File 1 Experimental details and characterization data.
