# Supplementary material for: Synthesis of 2H-furo[2,3-c]pyrazole ring systems through silver(I) ion-mediated ring-closure reaction
Source: Beilstein J Org Chem. 2019 Mar 14;15:679–84. doi: 10.3762/bjoc.15.62 (PMC6423569; doi:10.3762/bjoc.15.62)

BEILSTEIN JOURNAL OF ORGANIC CHEMISTRY

Supporting Information

for

Synthesis of 2*H*-furo[2,3-*c*]pyrazole ring systems through silver(I) ion-mediated ring-closure reaction

Vaida Milišiūnaitė, Rūta Paulavičiūtė, Eglė Arbačiauskienė, Vytas Martynaitis, Wolfgang Holzer and Algirdas Šačkus

Beilstein J. Org. Chem. **2019,** *15,* 679–684. doi:10.3762/bjoc.15.62

Experimental details and characterization data

License and Terms: This is a supporting information file under the terms of the Creative Commons Attribution License (http://creativecommons.org/licenses/by/4.0). Please note that the reuse, redistribution and reproduction in particular requires that the authors and source are credited.
The license is subject to the *Beilstein Journal of Organic Chemistry* terms and conditions: (https://www.beilstein-journals.org/bjoc)

General information

For thin-layer chromatography (TLC), Merck pre-coated TLC plates (silica gel 60 F_{254}) were employed. The purification of the products was performed using column chromatography with silica gel (high-purity grade 9385, pore size 60 Å, 230–400 mesh particle size). The melting points were determined in capillary tubes using a capillary melting point apparatus (Electrothermal MEL-TEMP®) and are uncorrected. The ^{1}H, ^{13}C and ^{15}N NMR spectra were recorded in $CDCl_3$ solutions at 25 °C on a Bruker Avance III 700 (700 MHz for ^{1}H, 176 MHz for ^{13}C, 71 MHz for ^{15}N) spectrometer equipped with a 5 mm TCI ^{1}H-^{13}C/^{15}N/D z-gradient cryoprobe. The chemical shifts, expressed in ppm, are reported relative to tetramethylsilane (TMS). The ^{15}N NMR spectra are referenced to neat, external nitromethane (coaxial capillary). The complete and unambiguous assignments of the ^{1}H, ^{13}C and ^{15}N resonances were achieved using standard Bruker software in conjunction with standard NMR spectroscopic techniques, such as DEPT, COSY, TOCSY, NOESY, gs-HSQC and gs-HMBC. The infrared spectra were recorded on a Bruker Vertex v70 FTIR spectrometer equipped with a diamond ATR accessory. HRMS spectra were recorded with a Bruker micrOTOF-QIII spectrometer. Starting materials and catalysts were purchased from Sigma-Aldrich and Fluorochem and used as received without further purification. Compound **3a** was prepared in accordance with the procedure reported elsewhere [1].

General procedure for the preparation of 4-alkynyl-1-phenyl-1*H*-pyrazol-3-ols 3b–j

To a solution of 4-iodo-1-phenyl-1*H*-pyrazol-3-ol (**2**, 1 mmol) in dry DMF (2 mL) under an argon atmosphere was added TEA (0.7 mL, 5 mmol), the appropriate ethyne (1.5 mmol), $Pd(PPh_3)_2Cl_2$ (70 mg, 0.1 mmol) and CuI (36 mg, 0.2 mmol). The mixture was stirred under an argon atmosphere at 58 °C for the given time, and subsequently diluted with water and extracted with ethyl acetate. The combined organic layers were washed with brine and dried over Na_2SO_4, and the solvent was evaporated. The residue was purified by flash chromatography (SiO_2, eluent: ethyl acetate/*n*-hexane 1:6, v/v) to yield compounds **3b-j**.

*4-[(4-Methylphenyl)ethynyl]-1-phenyl-1H-pyrazol-3-ol (**3b**)*

Reaction mixture was stirred for 12 h. Yield 206 mg (75%), white crystals. R_f = 0.27 (*n*-hexane/ethyl acetate 4/1, v/v), mp 168−169 °C (ethyl acetate). IR (ν_{max}, cm^{-1}): 3125, 3069, 3029 (OH, CH_{arom}), 2923 (CH_{aliph}), 2217 (C≡C), 1597, 1532, 1504, 1314, 1208 (C=C, C–N, C–O), 815, 755, 688 (CH=CH of mono- and disubstituted benzenes). ^{1}H NMR (700 MHz, $CDCl_3$): δ ppm 2.36 (CH_3), 7.14–7.15 (m, 2H, CPh 3,5-H), 7.29–7.31 (m, 1H, NPh 4-H),

7.42–7.43 (m, 2H, CPh 2,6-H), 7.48–7.50 (m, 2H, NPh 3,5-H), 7.53–7.54 (m, 2H, NPh 2,6-H), 7.87 (s, 1H, 5-H), 11.74 (br s, 1H, OH). ^{13}C NMR (176 MHz, $CDCl_3$): δ ppm 21.6 (CH_3), 77.8 (*C*H≡CPh), 92.6 (C-4), 92.8 (C≡*C*Ph), 119.2 (NPh C-2,6), 120.4 (CPh C-1), 126.8 (NPh C-4), 129.2 (CPh C-3,5), 130.0 (NPh C-3,5), 131.1 (C-5), 131.6 (CPh C-2,6), 138.3 (CPh C-4), 139.1 (NPh C-1), 163.8 (C-3). HRMS (ESI TOF): $[M+Na]^+$, found 297.0998. $[C_{18}H_{14}N_2O+Na]^+$ requires 297.0998.

*4-[(4-Ethylphenyl)ethynyl]-1-phenyl-1H-pyrazol-3-ol (**3c**)*

Reaction mixture was stirred for 3 h. Yield 225 mg (78%), yellowish crystals, R_f = 0.24 (*n*-hexane/ethyl acetate 4/1, v/v), mp 164−165 °C (ethyl acetate). IR (ν_{max}, cm^{-1}): 3060, 3029 (OH, CH_{arom}), 2964, 2928 (CH_{aliph}), 2220 (C≡C), 1597, 1540, 1503, 1414, 1208 (C=C, C–N, C–O), 830, 751, 678 (CH=CH of mono- and disubstituted benzenes). ^{1}H NMR (700 MHz, $CDCl_3$): δ ppm 1.25 (t, J = 7.7 Hz, 3H, CH_3), 2.66 (q, J = 7.7 Hz, 2H, CH_2), 7.18−7.18 (m, 2H, CPh 3,5-H), 7.29−7.31 (m, 1H, NPh 4-H), 7.45−7.50 (m, 4H, CPh 2,6-H, NPh 3,5-H), 7.53−7.54 (m, 2H, NPh 2,6-H), 7.87 (s, 1H, 5-H), 11.91 (br s, 1H, OH). ^{13}C NMR (176 MHz, $CDCl_3$): δ ppm 15.5 (CH_3), 28.9 (CH_2), 77.8 (*C*≡CPh), 92.6 (C-4), 92.8 (C≡*C*Ph), 119.2 (NPh C-2,6), 120.7 (CPh C-1), 126.8 (NPh C-4), 128.0 (CPh C-3,5), 130.0 (NPh C-3,5), 131.2 (C-5), 131.6 (CPh C-2,6), 139.0 (CPh C-4), 144.6 (NPh C-1), 163.9 (C-3). HRMS (ESI TOF): $[M+Na]^+$, found 311.1155. $[C_{19}H_{16}N_2O+Na]^+$ requires 311.1155.

*4-[(4-Fluorophenyl)ethynyl]-1-phenyl-1H-pyrazol-3-ol (**3d**)*

Reaction mixture was stirred for 12 h. Yield 203 mg (73%), white crystals, R_f = 0.35 (*n*-hexane/ethyl acetate 4/1, v/v), mp 197−198 °C (ethyl acetate). IR (ν_{max}, cm^{-1}): 3067, 3052 (OH, CH_{arom}), 2956, 2923 (CH_{aliph}), 2218 (C≡C), 1597, 1536, 1501, 1212 (C=C, C–N, C–O), 831, 754, 679 (CH=CH of mono- and disubstituted benzenes). ^{1}H NMR (700 MHz, $CDCl_3$): δ ppm 7.03 (t, J = 8.5 Hz, 2H, 4FPh 3,5-H), 7.30−7.32 (m, 1H, NPh 4-H), 7.48−7.52 (m, 4H, NPh 3,5-H, 4FPh 2,6-H), 7.53−7.54 (m, 2H, NPh 2,6-H), 7.88 (s, 1H, 5-H), 11.11 (br s, 1H, OH). ^{13}C NMR (176 MHz, $CDCl_3$): δ ppm 79.2 (*C*≡C4FPh), 91.6 (C≡*C*4FPh), 92.2 (C-4), 115.7 (^2J = 22.1 Hz, 4FPh C-3,5), 119.2 (NPh C-2,6), 119.6 (^4J = 3.8 Hz, 4FPh C-1), 126.6 (NPh C-4), 130.0 (NPh C-3,5), 131.1 (C-5), 133.5 (^3J = 8.9 Hz, 4FPh C-2,6), 139.1 (NPh C-1), 162.6 (J = 248.5 Hz, 4FPh C-4), 163.6 (C-3). HRMS (ESI TOF): $[M+Na]^+$, found 301.0748. $[C_{17}H_{11}FN_2O+Na]^+$ requires 301.0748.

*4-[(4-Etoxyphenyl)ethynyl]-1-phenyl-1H-pyrazol-3-ol (**3e**)*

Reaction mixture was stirred for 12 h. Yield 240 mg (79%), white crystals, R_f = 0.33 (*n*-hexane/ethyl acetate 4/1, v/v), mp 194−195 °C (ethyl acetate). IR (ν_{max}, cm^{-1}): 3056 (OH, CH_{arom}), 2977, 2923 (CH_{aliph}), 2134 (C≡C), 1597, 1502, 1473, 1245, 1173, 1043 (C=C, C–N, C–O), 824, 754, 678 (CH=CH of mono- and disubstituted benzenes). ^1H NMR (700 MHz, $CDCl_3$): δ ppm 1.42 (t, J = 7.0 Hz, 3H, CH_3), 4.05 (q, J = 7.0 Hz, 2H, CH_2), 6.85−6.86 (m, 2H, CPh 3,5-H), 7.28−7.30 (m, 1H, NPh 4-H), 7.45−7.46 (m, 2H, CPh 2,6-H), 7.47−7.49 (m, 2H, NPh 3,5-H), 7.53–7.55 (m, 2H, NPh 2,6-H), 7.86 (s, 1H, 5-H), 10.74 (br s, 1H, OH). ^{13}C NMR (176 MHz, $CDCl_3$): δ ppm 14.9 (CH_3), 63.6 (CH_2), 76.8 (*C*≡CPh), 92.6 (C-4), 92.7 (C≡*C*Ph), 114.6 (CPh C-3,5), 115.4 (CPh C-1), 119.0 (NPh C-2,6), 126.7 (NPh C-4), 129.9 (NPh C-3,5), 130.7 (C-5), 133.2 (CPh C-2,6), 139.2 (NPh C-1), 159.1 (CPh C-4), 163.5 (C-3). HRMS (ESI TOF): $[M+Na]^+$, found 327.1105. $[C_{19}H_{16}N_2O_2+Na]^+$ requires 327.1104.

*1-Phenyl-4-[(thiophen-3-yl)ethynyl]-1H-pyrazol-3-ol (**3f**)*

Reaction mixture was stirred for 2 h. Yield 197 mg (75%), yellowish crystals, R_f = 0.25 (*n*-hexane/ethyl acetate 4/1, v/v), mp 204.5−205.5 °C (ethyl acetate). IR (ν_{max}, cm^{-1}): 3127, 3109, 3069, 3059, 3046, 3004 (OH, CH_{arom}), 2220 (C≡C), 1595, 1533, 1503, 1397, 1305, 1254, 1206, 1060 (C=C, C–N, C–O), 751, 681 (CH=CH of monosubstituted benzene). ^1H NMR (700 MHz, DMSO-d_6): δ ppm 7.21 (dd, ^3J(4-H,5-H) = 5.0 Hz, ^4J(4-H,2-H) = 1.2 Hz, 1H, Th 4-H), 7.23–7.25 (m, 1H, Ph 4-H), 7.44–7.47 (m, 2H, Ph 3,5-H), 7.62 (dd, ^3J(5-H,4-H) = 4.9 Hz, ^4J(5-H,2-H) = 2.9 Hz, 1H, Th 5-H), 7.71−7.72 (m, 2H, Ph 2,6-H), 7.79 (dd, ^4J(2-H,5-H) = 3.0 Hz, ^4J(2-H,4-H) = 1.2 Hz, 1H, Th 2-H), 8.62 (s, 1H, 5-H), 11.07 (br s, 1H, OH). ^{13}C NMR (176 MHz, DMSO-d_6): δ ppm 79.6 (*C*≡CTh), 87.0 (C≡*C*Th), 91.3 (C-4), 117.2 (Ph C-2,6), 121.8 (Th C-3), 125.8 (Ph C-4), 126.8 (Th C-5), 128.9 (Th C-2), 129.5 (Ph C-3,5, Th C-4), 130.7 (C-5), 139.1 (Ph C-1), 162.7 (C-3). HRMS (ESI TOF): $[M+H]^+$, found 267.0584. $[C_{15}H_{10}N_2OS+H]^+$ requires 267.0587.

*4-(Hex-1-yn-1-yl)-1-phenyl-1H-pyrazol-3-ol (**3g**)*

Reaction mixture was stirred for 24 h. Yield 204 mg (85%), yellow crystals. R_f = 0.24 (*n*-hexane/ethyl acetate 4/1, v/v), mp 140−141.5 °C (ethyl acetate). IR (ν_{max}, cm^{-1}): 3120, 3070, 3049, 2958, 2931 (OH, CH_{arom}, CH_{aliph}), 1597, 1587 1526, 1504, 1415, 1307, 1234, 1210,

1062 (C=C, C–N, C–O), 756, 678 (CH=CH of monosubstituted benzene). ^{1}H NMR (700 MHz, $CDCl_3$): *δ* ppm 0.94 (t, *J* = 7.4 Hz, 3H, CH_3), 1.47 (sext, *J* = 7.4 Hz, 2H, CH_2CH_3), 1.59 (quin, *J* = 7.4 Hz, 2H, $CH_2C_2H_5$), 2.42 (t, *J* = 7.2 Hz, 2H, $CH_2C_3H_7$), 7.26−7.28 (m, 1H, Ph 4-H), 7.44−7.46 (m, 2H, Ph 3,5-H), 7.48−7.50 (m, 2H, Ph 2,6-H), 7.74 (s, 1H, 5-H), 11.65 (br s, 1H, OH). ^{13}C NMR (176 MHz, $CDCl_3$): *δ* ppm 13.8 (CH_3), 19.5 ($CH_2C_3H_7$), 22.2 (CH_2CH_3), 31.0 ($CH_2C_2H_5$), 69.1 (*C*≡C^{n}Bu), 92.9 (C-4), 93.8 (C≡*C*^{n}Bu), 119.0 (Ph C-2,6), 126.5 (Ph C-1), 129.9 (Ph C-3,5), 130.9 (C-5), 139.2 (Ph C-4), 163.9 (C-3). HRMS (ESI TOF): $[M+H]^+$, found 241.1332. $[C_{15}H_{16}N_2O+H]^+$ requires 241.1335.

*4-(Hept-1-yn-1-yl)-1-phenyl-1H-pyrazol-3-ol (**3h**)*

Reaction mixture was stirred for 12 h. Yield 198 mg (78%), yellowish crystals. R_f = 0.25 (*n*-hexane/ethyl acetate 4/1, v/v), mp 110−111 °C (ethyl acetate). IR (ν_{max}, cm^{-1}): 3131, 3057 (OH, CH_{arom}), 2947, 2922 (CH_{aliph}), 2163 (C≡C), 1595, 1529, 1499 (C=C, C–N, C–O), 761, 691 (CH=CH of monosubstituted benzene). ^{1}H NMR (700 MHz, $CDCl_3$): *δ* ppm 0.91 (t, *J* = 7.3 Hz, 3H, CH_3), 1.35 (sext, *J* = 7.0 Hz, 2H, CH_2CH_3), 1.42 (quin, *J* = 7.0 Hz, 2H, $CH_2C_2H_5$), 1.61 (quin, *J* = 7.3 Hz, 2H, $CH_2C_3H_7$), 2.42 (t, *J* = 7.2 Hz, 2H, $CH_2C_4H_9$), 7.26−7.28 (m, 1H, Ph 4-H), 7.44−7.46 (m, 2H, Ph 3,5-H), 7.49−7.50 (m, 2H, Ph 2,6-H), 7.74 (s, 1H, 5-H), 11.41 (br s, 1H, OH). ^{13}C NMR (176 MHz, $CDCl_3$): *δ* ppm 14.2 (CH_3), 19.8 ($CH_2C_4H_9$), 22.4 (CH_2CH_3), 28.6 ($CH_2C_3H_7$), 31.3 ($CH_2C_2H_5$), 69.1 (*C*≡C^{n}Pent), 92.8 (C-4), 93.9 (C≡*C*^{n}Pent), 119.0 (Ph C-2,6), 126.5 (Ph C-4), 129.9 (Ph C-3,5), 130.9 (C-5), 139.2 (Ph C-4), 163.8 (C-3). HRMS (ESI TOF): $[M+Na]^+$, found 277.1310. $[C_{16}H_{18}N_2O+Na]^+$ requires 277.1311.

*4-(Cyclopropylethynyl)-1-phenyl-1H-pyrazol-3-ol (**3j**)*

Reaction mixture was stirred for 2 h. Yield 143 mg (64%), white crystals. R_f = 0.23 (*n*-hexane/ethyl acetate 4/1, v/v), mp 185.5−187 °C (ethyl acetate). IR (ν_{max}, cm^{-1}): 3122, 3074, 3051, 2999, 2954, 2920 (OH, CH_{arom}, CH_{aliph}), 1596, 1526, 1504, 1420, 1308, 1242, 1210, 1063(C=C, C–N, C–O), 758, 678 (CH=CH of monosubstituted benzene). ^{1}H NMR (700 MHz, $CDCl_3$): *δ* ppm 0.79−0.81 (m, 2H, CH_2), 0.82−0.86 (m, 2H, CH_2), 1.45−1.49 (m, 1H, $CH(CH_2)_2$), 7.26−7.28 (m, 1H, Ph 4-H), 7.44−7.48 (m, 4H, Ph 2,3,5,6-H), 7.72 (s, 1H, 5-H), 11.45 (br s, 1H, OH). ^{13}C NMR (176 MHz, $CDCl_3$): *δ* ppm 0.6 ($CH(CH_2)_2$), 8.8 ($CH(CH_2)_2$), 63.8 (*C*≡CC_3H_5), 92.2 (C-4), 96.2 (C≡*C*C_3H_5), 118.5 (Ph C-2,6), 126.0 (Ph C-4), 129.4 (Ph C-

3,5), 130.5 (C-5), 138.6 (Ph C-1), 163.4 (C-3). HRMS (ESI TOF): $[M+H]^+$, found 225.1021. $[C_{14}H_{12}N_2O+H]^+$ requires 225.1022.

General procedure for the preparation of 5-substituted 2*H*-furo[2,3-*c*]pyrazoles 4a–j

To a solution of appropriate pyrazole **3** (0.5 mmol) in absolute DMF (1 mL), AgOTf (13 mg, 10 mol %) and K_2CO_3 (110 mg, 1 mmol) were added. The mixture was stirred at 120 °C for 14 hours, diluted with water and the extraction was done with ethyl acetate. The combined organic layers were washed with brine, dried over Na_2SO_4, the solvent was evaporated. The residue was purified by flash chromatography (SiO_2, eluent: ethyl acetate/*n*-hexane 1:7, v/v) to yield compounds **4a**–**j**.

*2,5-Diphenyl-2H-furo[2,3-c]pyrazole (**4a**)*

Yield 112 mg (92%), yellowish crystals. R_f = 0.47 (*n*-hexane/ethyl acetate 4/1, v/v), mp 193.5–194.3°C (ethyl acetate). IR (ν_{max}, cm^{-1}): 3139, 3062, 3045 (CH_{arom}), 1622, 1596, 1587, 1511, 1481, 1454, 1434, 1388, 1315, 1271, 1198, 1154, 1073, 1035, 1004 (C=C, C–N, C–O), 748, 722, 685 (CH=CH of monosubstituted benzenes). ^{1}H NMR (700 MHz, $CDCl_3$): δ ppm 6.74 (s, 1H, 4-H), 7.27−7.29 (m, 1H, NPh 4-H), 7.33−7.35 (m, 1H, CPh 4-H), 7.42−7.45 (m, 2H, CPh 3,5-H), 7.45−7.48 (m, 2H, NPh 3,5-H), 7.72−7.73 (m, 2H, NPh 2,6-H), 7.77 (s, 1H, 3-H), 7.79−7.80 (m, 2H, CPh 2,6-H). ^{13}C NMR (176 MHz, $CDCl_3$): δ ppm 96.4 (C-4), 113.8 (C-3a), 116.3 (C-3), 119.2 (NPh C-2,6), 124.6 (CPh C-2,6), 126.3 (NPh C-4), 128.6 (CPh C-4), 128.9 (CPh C-3,5), 129.6 (NPh C-3,5), 130.7 (CPh C-1), 141.1 (NPh C-1), 159.3 (C-5), 169.0 (C-6a). HRMS (ESI TOF): $[M+Na]^+$, found 283.0842. $[C_{17}H_{12}N_2O+Na]^+$ requires 283.0842.

*5-(4-Methylphenyl)-2-phenyl-2H-furo[2,3-c]pyrazole (**4b**)*

Yield 119 mg (87%), white crystals. R_f = 0.48 (*n*-hexane/ethyl acetate 4/1, v/v), mp 211−212°C (ethyl acetate). IR (ν_{max}, cm^{-1}): 3045, 3020 (CH_{arom}), 2956, 2917 (CH_{aliph}), 1694, 1597, 1500, 1441, 1388 (C=C, C–N, C–O), 796, 746, 684 (CH=CH of mono- and disubstituted benzenes). ^{1}H NMR (700 MHz, $CDCl_3$): δ ppm 2.39 (CH_3), 6.68 (s, 1H, 4-H), 7.23−7.25 (m, 2H, 5-Ph 3,5-H), 7.26−7.28 (m, 1H, NPh 4-H), 7.45−7.47 (m, 2H, NPh 3,5-H), 7.67−7.69 (m, 2H, 5-Ph 2,6-H), 7.71−7.73 (m, 2H, NPh 2,6-H), 7.74 (s, 1H, 3-H). ^{13}C NMR

(176 MHz, $CDCl_3$): δ ppm 21.5 (CH_3), 95.6 (C-4), 113.8 (C-3a), 116.1 (C-3), 119.1 (NPh C-2,6), 124.6 (5-Ph C-2,6), 126.2 (NPh C-4), 128.0 (5-Ph C-1), 129.6 (NPh C-3,5), 129.6 (5-Ph C-3,5), 138.7 (5-Ph C-4), 141.1 (NPh C-1), 159.6 (C-5), 168.9 (C-6a). ^{15}N NMR (71 MHz, $CDCl_3$): δ ppm −169.9 (N-2), −127.9 (N-1). HRMS (ESI TOF): $[M+Na]^+$, found 297.0999. $[C_{18}H_{14}N_2O+Na]^+$ requires 297.0998.

*5-(4-Ethylphenyl)-2-phenyl-2H-furo[2,3-c]pyrazole (**4c**)*

Yield 133 mg (92%), white crystals. R_f = 0.51 (*n*-hexane/ethyl acetate 4/1, v/v), mp 180−181 °C (ethyl acetate). IR (ν_{max}, cm^{-1}): 3060 (CH_{arom}), 2965, 2921 (CH_{aliph}), 1618, 1597, 1501, 1480, 1447, 1386 (C=C, C–N, C–O), 834, 753, 687 (CH=CH of mono- and disubstituted benzenes). ^1H NMR (700 MHz, $CDCl_3$): δ ppm 1.25 (t, J = 7.6 Hz, 3H, CH_3), 2.67 (q, J = 7.6 Hz, 2H, CH_2), 6.66 (s, 1H, 4-H), 7.24−7.26 (m, 3H, 5-Ph 3,5-H, NPh 4-H), 7.43−7.45 (m, 2H, NPh 3,5-H), 7.68−7.71 (m, 4H, 5-Ph 2,6-H, NPh 2,6-H), 7.72 (s, 1H, 3-H). ^{13}C NMR (176 MHz, $CDCl_3$): δ ppm 15.6 (CH_3), 28.9 (CH_2), 95.6 (C-4), 113.8 (C-3a), 116.1 (C-3), 119.1 (NPh C-2,6), 124.7 (5-Ph C-2,6), 126.2 (NPh C-4), 128.2 (5-Ph C-1), 128.4 (5-Ph C-3,5), 129.6 (NPh C-3,5), 141.1 (NPh C-1), 145.1 (5-Ph C-4), 159.6 (C-5), 168.9 (C-6a). ^{15}N NMR (71 MHz, $CDCl_3$): δ ppm −170.0 (N-2), −128.0 (N-1). HRMS (ESI TOF): $[M+Na]^+$, found 311.1156. $[C_{19}H_{16}N_2O+Na]^+$ requires 311.1155.

*5-(4-Fluorophenyl)-2-phenyl-2H-furo[2,3-c]pyrazole (**4d**)*

Yield 120 mg (86%), white crystals. R_f = 0.48 (*n*-hexane/ethyl acetate 4/1, v/v), mp 220−221 °C (ethyl acetate). IR (ν_{max}, cm^{-1}): 3066, 3047 (CH_{arom}), 2955, 2921 (CH_{aliph}), 1596, 1498, 1447, 1385, 1223 (C=C, C–N, C–O), 843, 750, 687 (CH=CH of mono- and disubstituted benzenes). ^1H NMR (700 MHz, $CDCl_3$): δ ppm 6.67 (s, 1H, 4-H), 7.13 (t, J = 8.5 Hz, 2H, 4FPh 3,5-H), 7.27−7.29 (m, 1H, NPh 4-H), 7.45−7.48 (m, 2H, NPh 3,5-H), 7.71−7.73 (m, 2H, NPh 2,6-H), 7.75−7.77 (m, 3H, 4FPh 2,6-H, 3-H). ^{13}C NMR (176 MHz, $CDCl_3$): δ ppm 96.2 (C-4), 113.7 (C-3a), 116.1 (C-3), 116.2 (4FPh C-3,5, ^2J = 61.1 Hz), 119.2 (NPh C-2,6), 126.4 (NPh C-4), 126.5 (4FPh C-2,6, ^3J = 8.1 Hz), 127.0 (4FPh C-1, ^4J = 2.7 Hz), 129.6 (NPh C-3,5), 141.1 (NPh C-1), 158.3 (C-5), 162.9 (4FPh C-4, ^1J = 248.6 Hz), 168.9 (C-6a). ^{15}N NMR (71 MHz, $CDCl_3$): δ ppm −169.4 (N-2), −127.5 (N-1). HRMS (ESI TOF): $[M+Na]^+$, found 301.0749. $[C_{17}H_{11}FN_2O+Na]^+$ requires 301.0748.

*5-(4-Ethoxyphenyl)-2-phenyl-2H-furo[2,3-c]pyrazole (**4e**)*

Yield 123 mg (81%), white crystals. R_f = 0.53 (*n*-hexane/ethyl acetate 4/1, v/v), mp 190−191 °C (ethyl acetate). IR (ν_{max}, cm^{-1}): 3063, 3046 (CH_{arom}), 2978, 2924 (CH_{aliph}), 1597, 1501, 1387, 1249, 1239 (C=C, C–N, C–O). ^{1}H NMR (700 MHz, $CDCl_3$): δ ppm 1.44 (t, J = 7.0 Hz, 3H, CH_3), 4.08 (q, J = 7.0 Hz, 2H, CH_2), 6.58 (s, 1H, 4-H), 6.94−6.96 (m, 2H, 5-Ph 3,5-H), 7.25−7.27 (m, 1H, NPh 4-H), 7.44−7.47 (m, 2H, NPh 3,5-H), 7.69−7.72 (m, 4H, NPh 2,6-H, 5-Ph 2,6-H), 7.72 (s, 1H, 3-H). ^{13}C NMR (176 MHz, $CDCl_3$): δ ppm 14.9 (CH_3), 63.7 (CH_2), 94.5 (C-4), 113.9 (C-3a), 114.9 (5-Ph C-3,5), 115.8 (C-3), 119.1 (NPh C-2,6), 123.4 (5-Ph C-1), 126.09 (NPh C-4), 126.13 (5-Ph C-2,6), 129.6 (NPh C-3,5), 141.2 (NPh C-1), 159.46 (5-Ph C-4), 159.54 (C-5), 168.9 (C-6a). ^{15}N NMR (71 MHz, $CDCl_3$): δ ppm −170.6 (N-2), −128.2 (N-1). HRMS (ESI TOF): $[M+Na]^+$, found 327.1104. $[C_{19}H_{16}N_2O_2+Na]^+$ requires 327.1104.

*2-Phenyl-5-(thiophen-3-yl)-2H-furo[2,3-c]pyrazole (**4f**)*

Yield 83 mg (62%), yellowish crystals, R_f = 0.45 (*n*-hexane/ethyl acetate 4/1, v/v), mp 191−193 °C (ethyl acetate). IR (ν_{max}, cm^{-1}): 3139, 3128, 3110, 3097, 3065, 3045(CH_{arom}), 1594, 1587, 1510, 1497, 1480, 1463, 1441. 1386, 1328, 1239, 1218, 1177, 1075, 1036, 1026 (C=C, C–N, C–O), 777, 684 (CH=CH of monosubstituted benzene). ^{1}H NMR (700 MHz, $CDCl_3$): δ ppm 6.54 (s, 1H, 4-H), 7.26−7.28 (m, 1H, Ph 4-H), 7.37−7.39 (m, 2H, Th H), 7.44−7.47 (m, 2H, Ph 3,5-H), 7.66 (dd, ^4J(2-H,5-H) = 2.8 Hz, ^4J(2-H,4-H) = 1.4 Hz, 1H, Th 2-H), 7.71–7.72 (m, 2H, Ph 2,6-H), 7.74 (s, 1H, 3-H). ^{13}C NMR (176 MHz, $CDCl_3$): δ ppm 96.1 (C-4), 113.6 (C-3a), 116.3 (C-3), 119.1 (Ph C-2,6), 121.3 (Th C-2), 124.7 (Th C), 126.2 (Ph C-4), 126.7 (Th C), 129.6 (Ph C-3,5), 132.3 (Th C-3), 141.1 (Ph C-1), 156.1 (C-5), 168.7 (C-6a). HRMS (ESI TOF): $[M+H]^+$, found 267.0589. $[C_{15}H_{10}N_2OS+H]^+$ requires 267.0587.

*5-Butyl-2-phenyl-2H-furo[2,3-c]pyrazole (**4g**)*

Yield 113 mg (94%), yellowish crystals. R_f = 0.58 (*n*-hexane/ethyl acetate 4/1, v/v), mp 88.5–90 °C (ethyl acetate). IR (ν_{max}, cm^{-1}): 3141, 3126, 3074, 3050 (CH_{arom}), 2968, 2954, 2928, 2857 (CH_{aliph}), 1595, 1577, 1506, 1460, 1447, 1438, 1382, 1320, 1231, 1202, 1120, 1073, 1035 (C=C, C–N, C–O), 753, 691 (CH=CH of monosubstituted benzene). ^{1}H NMR (700 MHz, $CDCl_3$): δ ppm 0.96 (t, J = 7.4 Hz, 3H, CH_3), 1.42 (sext, J = 7.4 Hz, 2H,

CH_2CH$_3$), 1.71 (quin, J = 7.6 Hz, 2H, CH_2C$_2$H$_5$), 2.7 (t, J = 7.5 Hz, 2H, CH_2C$_3$H$_7$), 6.07 (s, 1H, 4-H), 7.23–7.25 (m, 1H, Ph 4-H), 7.42−7.44 (m, 2H, Ph 3,5-H), 7.62 (s, 1H, 3-H), 7.68−7.69 (m, 2H, Ph 2,4-H). ^{13}C NMR (176 MHz, CDCl$_3$): δ ppm 13.9 (CH$_3$), 22.3 (CH$_2$CH$_3$), 28.9 (CH$_2$C$_3$H$_7$), 29.7 (CH$_2$C$_2$H$_5$), 96.8 (C-4), 113.2 (C-3a), 115.2 (C-3), 119.0 (Ph C-2,6), 125.9 (Ph C-4), 129.5 (Ph C-3,5), 141.3 (Ph C-1), 163.2 (C-5), 169.0 (C-6a). HRMS (ESI TOF): [M+Na]$^+$, found 263.1152. [$C_{15}H_{16}N_2O$+Na]$^+$ requires 263.1155.

*5-Pentyl-2-phenyl-2H-furo[2,3-c]pyrazole (**4h**)*

Yield 104 mg (82%), white crystals. R$_f$ = 0.54 (n-hexane/ethyl acetate 4/1, v/v), mp 77-78 °C (ethyl acetate). IR (ν_{max}, cm^{-1}): 3068, 3048 (CH$_{arom}$), 2952, 2926 (CH$_{aliph}$), 1599, 1580, 1508, 1448, 1388 (C=C, C–N, C–O), 727, 686 (CH=CH of monosubstituted benzene). ^{1}H NMR (700 MHz, CDCl$_3$): δ ppm 0.94 (t, J = 7.1 Hz, 3H, CH$_3$), 1.37−1.42 (m, 4H, C$_2$$H_4CH_3$), 1.75 (quin, J = 7.4 Hz, 2H, CH_2C$_3$H$_7$), 2.71 (t, J = 7.4 Hz, 2H, CH_2C$_4$H$_9$), 6.09 (s, 1H, 4-H), 7.25−7.28 (m, 1H, Ph 4-H), 7.44−7.46 (m, 2H, Ph 3,5-H), 7.65 (s, 1H, 3-H), 7.70−7.71 (m, 2H, Ph 2,4-H). ^{13}C NMR (176 MHz, CDCl$_3$): δ ppm 14.1 (CH$_3$), 22.5 (CH$_2$CH$_3$), 27.3 (CH$_2$C$_3$H$_7$), 29.1 (CH$_2$C$_4$H$_9$), 31.4 (CH$_2$C$_2$H$_5$), 96.8 (C-4), 113.1 (C-3a), 115.2 (C-3), 119.0 (Ph C-2,6), 125.9 (Ph C-4), 129.5 (Ph C-3,5), 141.3 (Ph C-1), 163.2 (C-5), 168.9 (C-6a). HRMS (ESI TOF): [M+Na]$^+$, found 277.1311. [$C_{16}H_{18}N_2O$+Na]$^+$ requires 277.1311.

*5-Cyclopropyl-2-phenyl-2H-furo[2,3-c]pyrazole (**4j**)*

Yield 101 mg (90%), yellowish crystals. R$_f$ = 0.60 (n-hexane/ethyl acetate 4/1, v/v), mp 98.5−100 °C (ethyl acetate). IR (ν_{max}, cm^{-1}): 3149, 3135, 3089, 3027, 3007 (CH$_{arom}$), 2955, 2917, 2850 (CH$_{aliph}$), 1595, 1585, 1507, 1460, 1443, 1390, 1374, 1296, 1230, 1213, 1122, 1045, 1035, (C=C, C–N, C–O), 757, 689 (CH=CH of monosubstituted benzene). ^{1}H NMR (700 MHz, CDCl$_3$): δ ppm 0.94−0.96 (m, 4H, CH(CH_2)$_2$), 1.93−1.97 (m, 1H, CH(CH$_2$)$_2$), 6.06 (s, 1H, 4-H), 7.22−7.24 (m, 1H, Ph 4-H), 7.41−7.44 (m, 2H, Ph 3,5-H), 7.60 (s, 1H, 3-H), 7.66−7.67 (m, 2H, Ph 2,6-H). ^{13}C NMR (176 MHz, CDCl$_3$): δ ppm 7.1 (CH(CH$_2$)$_2$), 10.1 (CH(CH$_2$)$_2$), 95.5 (C-4), 113.2 (C-3a), 115.0 (C-3), 119.0 (Ph C-2,6), 125.9 (Ph C-4), 129.5 (Ph C-3,5), 141.3 (Ph C-1), 163.8 (C-5), 168.4 (C-6a). HRMS (ESI TOF): [M+H]$^+$, found 225.1022 [$C_{14}H_{12}N_2O$+H]$^+$ requires 225.1022.

X-ray analysis of compound 4d

The X-ray intensity data was measured on a Bruker-Nonius KappaCCD diffractometer equipped with graphite monochromator, Mo Kα INCOATEC micro focus sealed tube and Cryostream 700 cooling device. The structure was solved by direct method and refined by full-matrix least-squares techniques. Non-hydrogen atoms were refined with anisotropic displacement parameters. Hydrogen atoms were fitted to the peaks of the difference synthesis as well as calculated geometrically and refined with a riding model. The following software was used: DENZO and HKL SCALEPEAK software package [2] using a narrow-frame algorithm for frame integration, no absorption correction due to small absorption coefficient, SIR-92 [3] for structure solution, refinement molecular diagrams and graphical user-interface, SHELXL [4] for refinement and graphical user-interface, Platon [5] for symmetry check and Cg(Pi-Ring) interactions calculations. Experimental data and CCDC-code can be found in Table S1. Crystal data, data collection parameters, and structure refinement details are given in Tables S2 and S3. Molecular structure in “Ortep View” is displayed in Figure S1 and arrangement of enantiomers in the crystal is displayed in Figure S2.

Table S1: Experimental parameter and CCDC-Code

**Sample**	**Machine**	**Source**	**Temp.**	**Detector distance**	**Time/Frame**	**#Frames**	**Frame width**	**CCDC**
			[K]	[mm]	[s]		[°]	
**4d**	Bruker-Nonius KappaCCD	MoK\a, fine-focus sealed tube	193	32	35	330	1.0	1862867

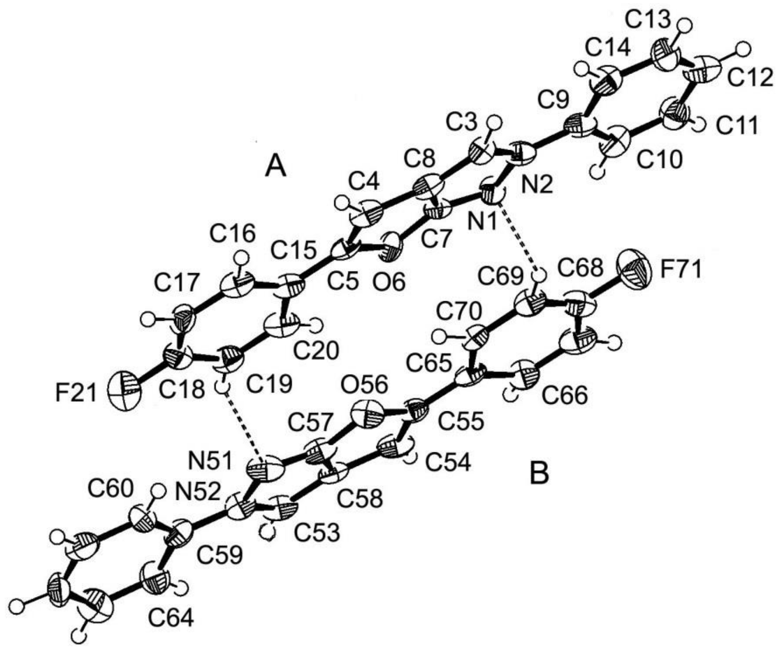

Figure S1: Asymmetric unit of compound **4d**.

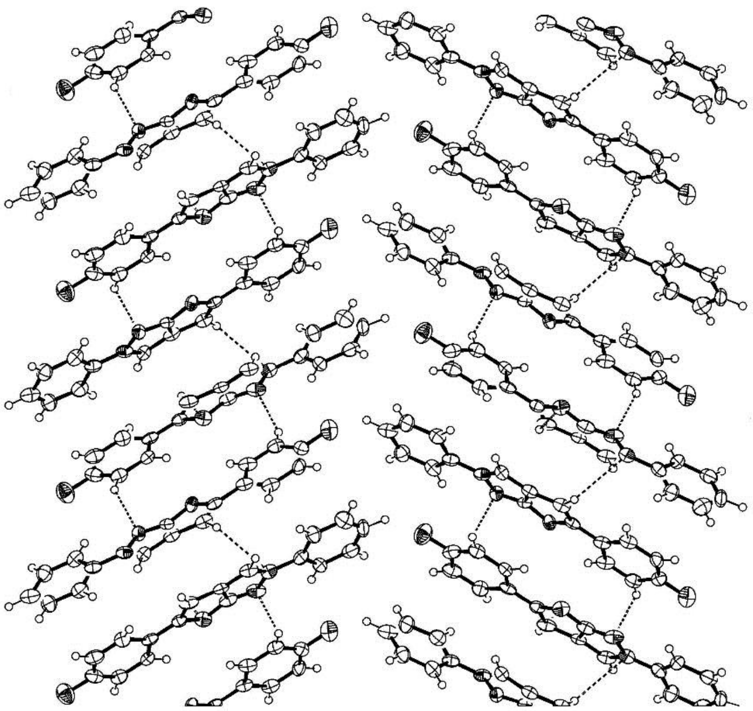

Figure S2: View (normal to (100)) of molecular packing in the crystal.

Table S2: Sample and crystal data of compound **4d**.

**Chemical formula**	$C_{17}H_{11}FN_2O$	**Crystal system**	Orthorhombic	
**Formula weight [g/mol]**	278.28	**Space group**	*P bn2₁*	
**Temperature [K]**	193	**Z**	8	
**Measurement method**	φ and ω scans	**Volume [Å^3]**	2568.4 (2)	
**Radiation wavelength**	0.71073	**Unit cell dimensions [Å] and [°]**	5.4969(3)	90

**[Å]**			16.2128(7)<br>28.819(2)	90<br>90
**Crystal size / [mm^3]**	0.21×0.12×0.01			
**Crystal habit**	Colourless plate			
**Density (calculated) / [g/cm^3]**	1.439	**Absorption coefficient / mm^{-1}]**	0.101	
**Abs. correction Tmin**	-	**Abs. correction Tmax**	-	
**Abs. correction type**	no absorption correction	**F(000) [e$^-$]**	1152	

Table S3: Data collection and structure refinement of compound **4d**.

**Index ranges**	$-7 \leq h \leq 7$<br>$-21 \leq k \leq 21$<br>$-38 \leq l \leq 38$	**Theta range for data collection [°]**	1.4-28.5	
**Reflections number**	4327	**Data / restraints / parameters**	2787/1/379	
**Refinement method**	Least-squares matrix: full	**Final R indices**	All data	R1 = 0.162 wR2 = 0.148
**Function minimized**	Σ w(F_0^2 - F_c^2)^2		I > 2σ(I)	R1 = 0.0739 wR2 = 0.121
**Goodness-of-fit on F^2**	1.056	**Weighting scheme**	$w = 1/[\sigma^2(F_o^2) + (0.0483P)^2 + 1.1838P]$ where $P = (F_o^2 + 2F_c^2)/3$	
**Largest diff. peak and hole [e Å^{-3}]**	-0.255 +0.234			

References

1. Arbačiauskienė, E.; Vilkauskaitė, G.; Eller, G. A.; Holzer, W.; Šačkus, A. *Tetrahedron* **2009**, *65*, 7817–7824.
2. Otwinowski, Z.; Minor, W. Processing of X-ray diffraction data collected in oscillation mode. In *Methods in Enzymology;* Carter, C. W. Jr.; Sweet, R. M., Eds.; Academic Press: New York, 1997; Vol. 276, pp. 307–326. doi:10.1016/S0076-6879(97)76066-X
3. Altomare, A.; Cascarano, G.; Giacovazzo, C.; Guagliardi, A.; Burla, M. C.; Polidori, G.; Camalli, M. *J. Appl. Crystallogr.* **1994**, *27*, 435–436. doi:10.1107/S0021889894000221
4. Sheldrick, G.M. *Acta Crystallogr.*, **2008**, *A64*, 112-122. doi: 10.1107/S0108767307043930

5. Spek, A. L. *Acta Crystallogr., Sect. D: Biol. Crystallogr.* **2009**, *65*, 148–155. doi:10.1107/S090744490804362X

^{1}H NMR and ^{13}C NMR spectra of compounds 4a–j

^{1}H NMR spectrum of 4a ($CDCl_3$, 700 MHz):

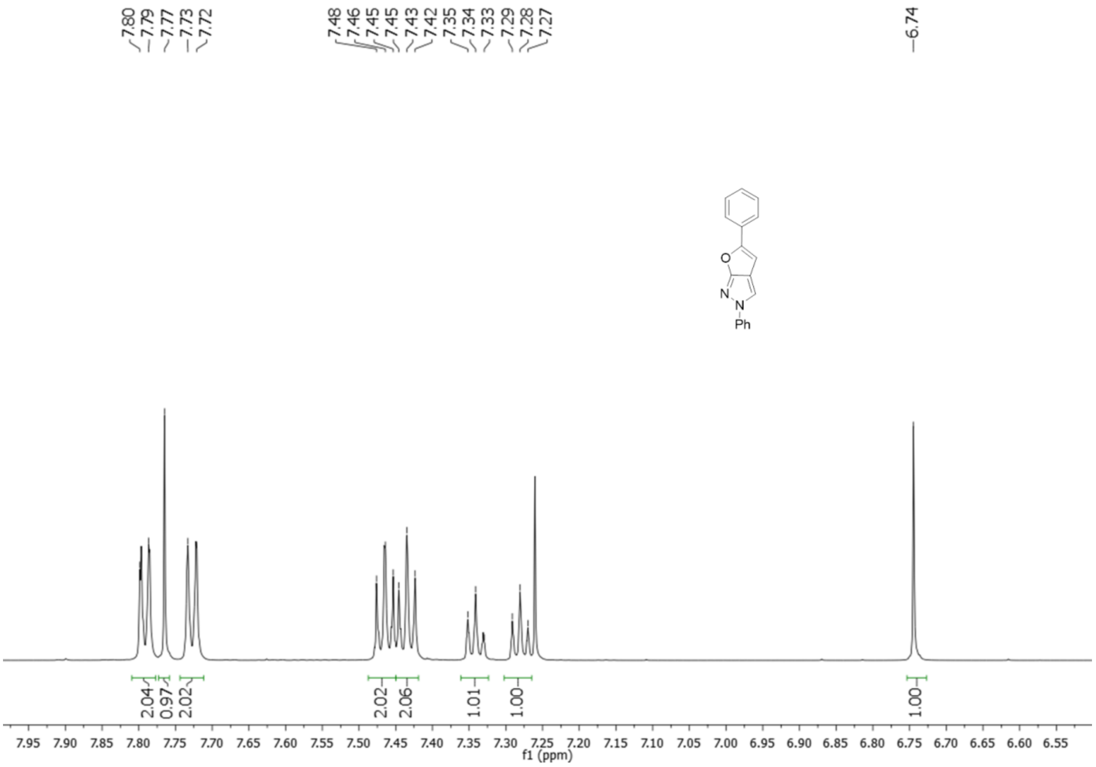

^{13}C NMR spectrum of 4a ($CDCl_3$, 176 MHz):

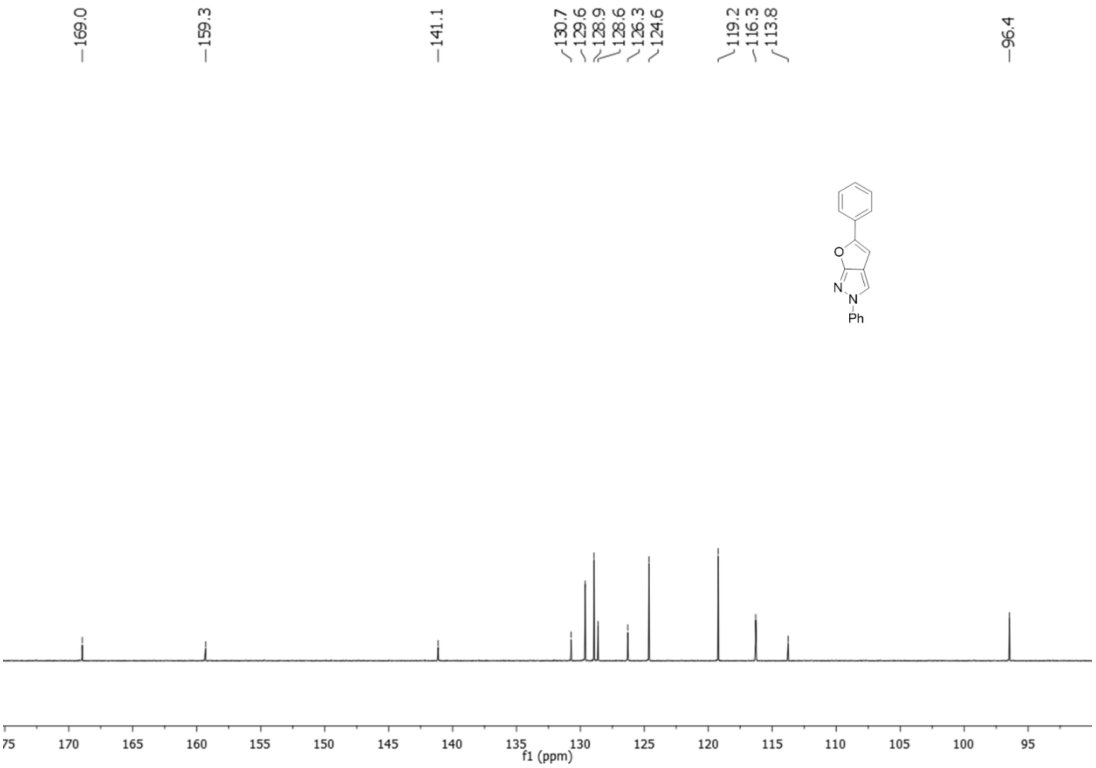

^{1}H NMR spectrum of 4b ($CDCl_3$, 700 MHz):

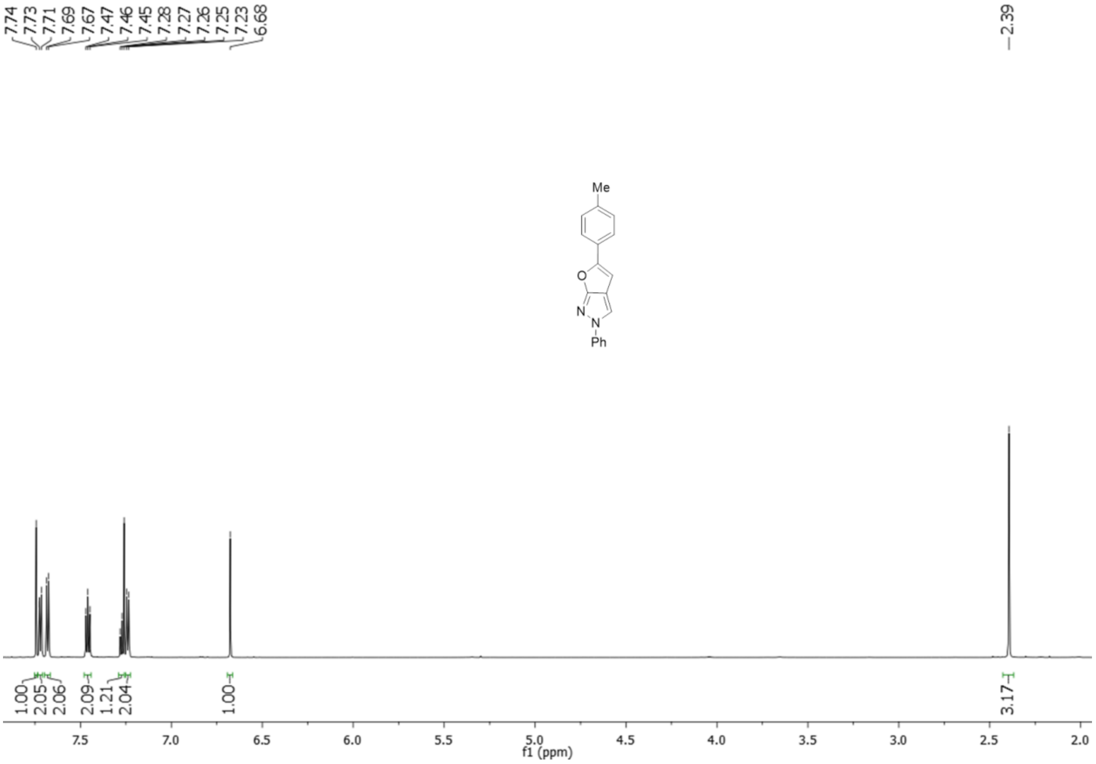

^{13}C NMR spectrum of 4b ($CDCl_3$, 176 MHz):

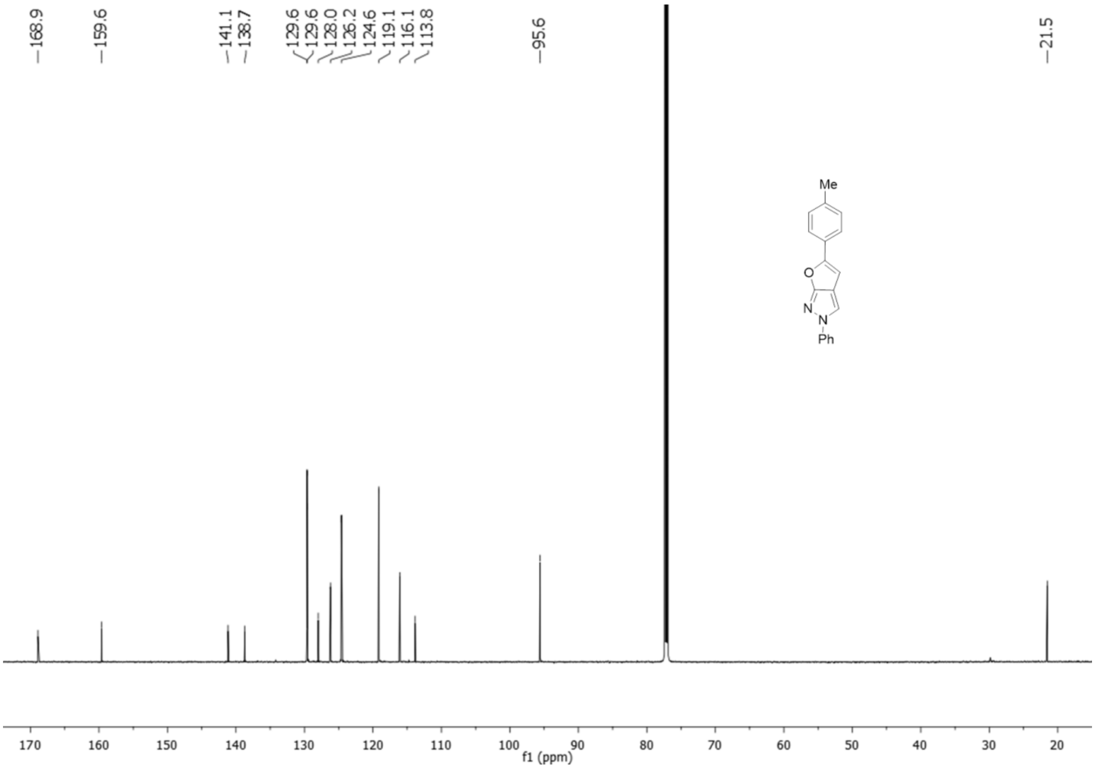

^{1}H NMR spectrum of 4c ($CDCl_3$, 700 MHz):

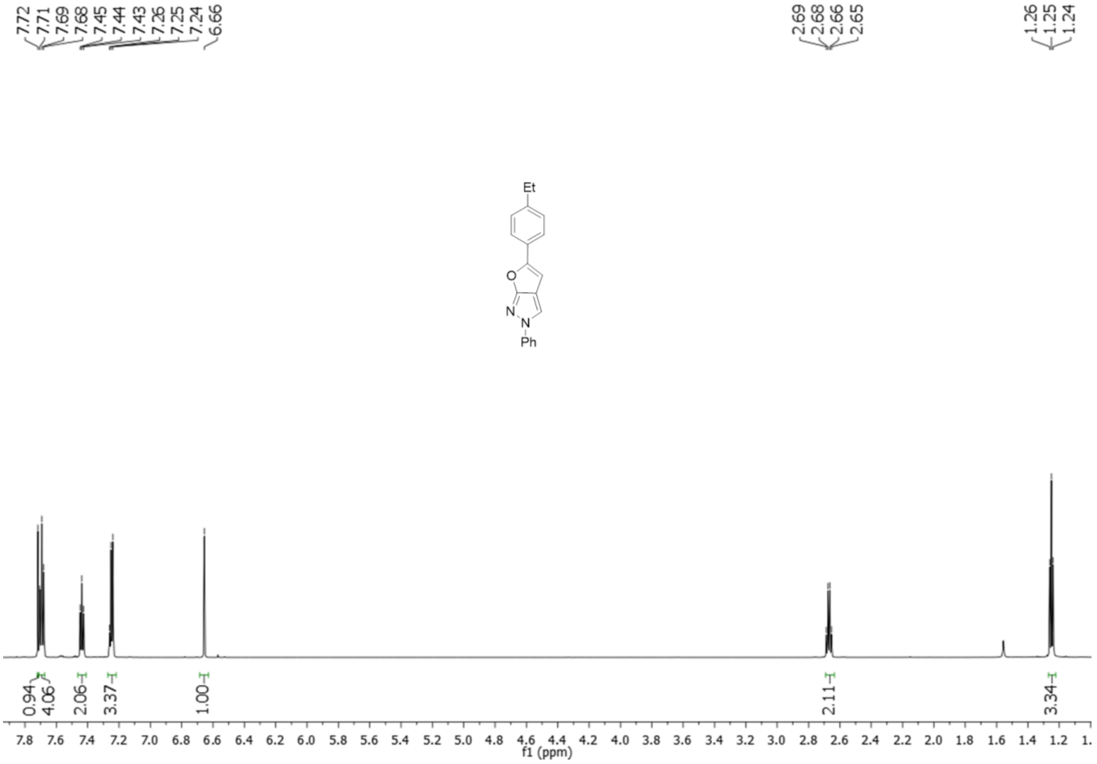

^{13}C NMR spectrum of 4c ($CDCl_3$, 176 MHz):

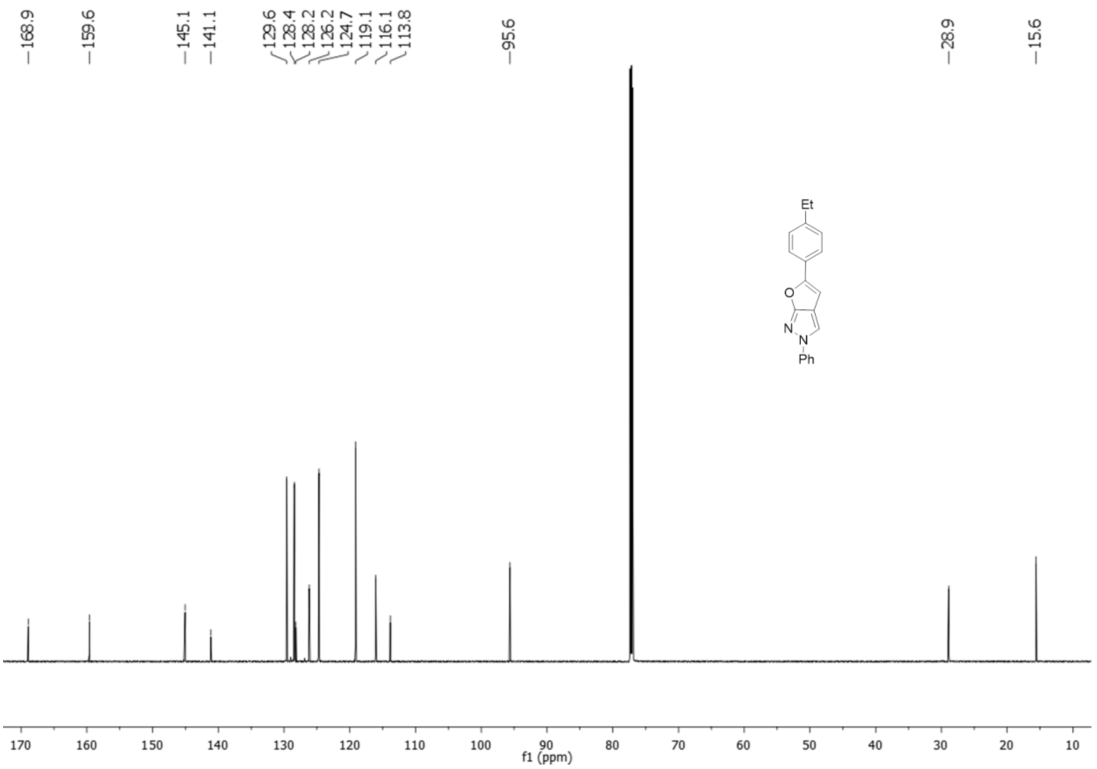

^{1}H NMR spectrum of 4d ($CDCl_3$, 700 MHz):

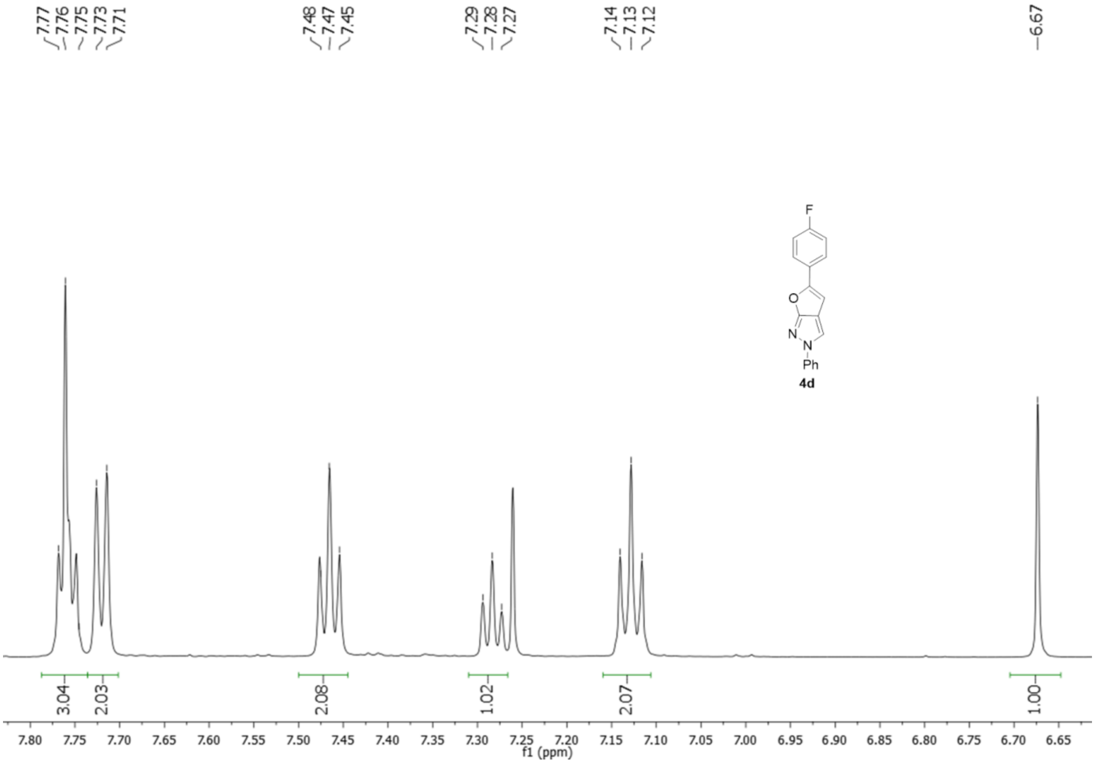

^{13}C NMR spectrum of 4d ($CDCl_3$, 176 MHz):

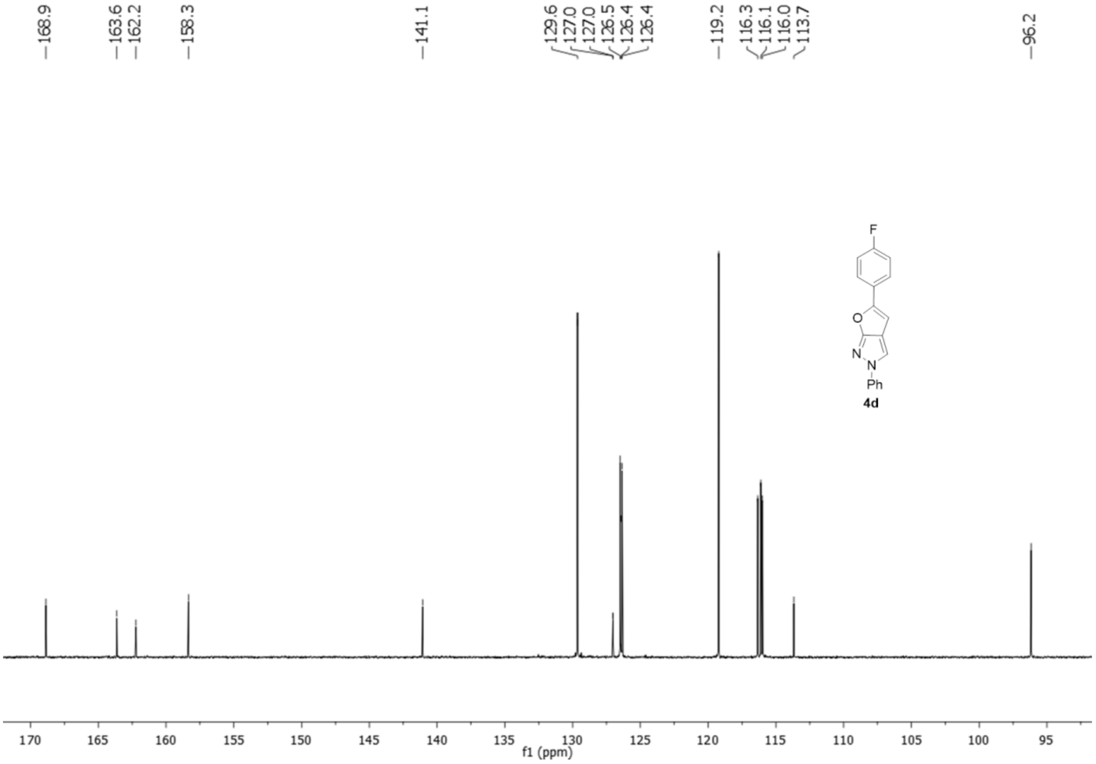

^{1}H NMR spectrum of 4e ($CDCl_3$, 700 MHz):

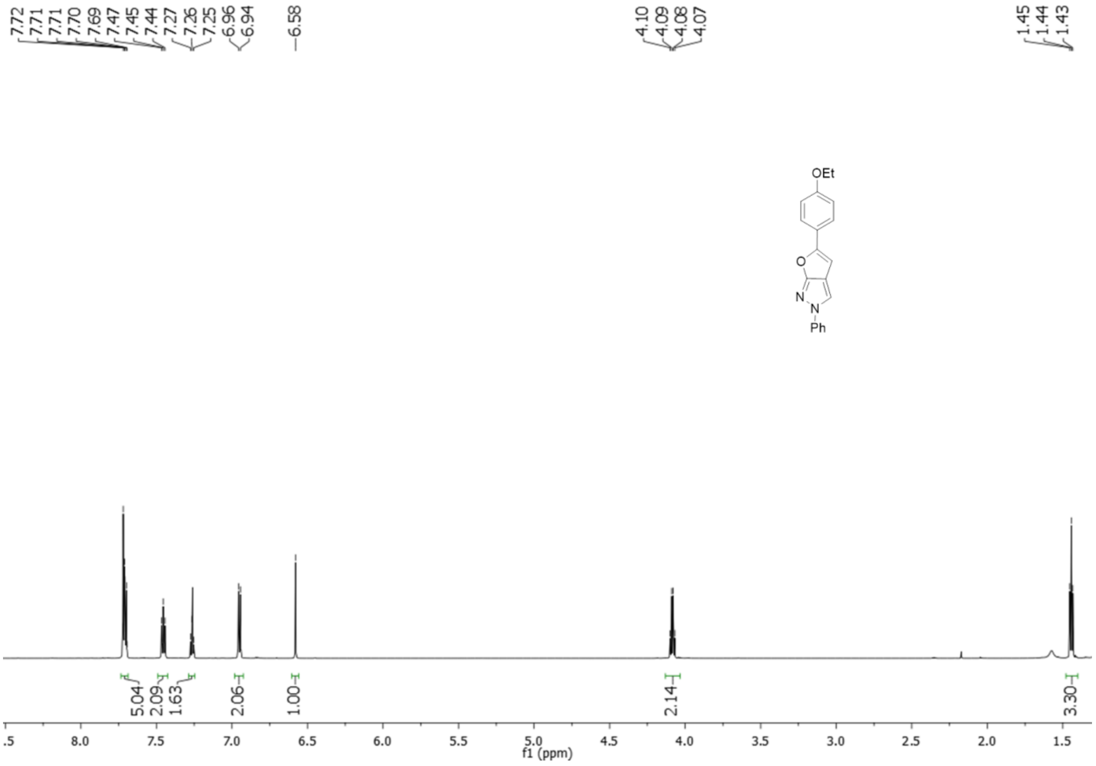

^{13}C NMR spectrum of 4e ($CDCl_3$, 176 MHz):

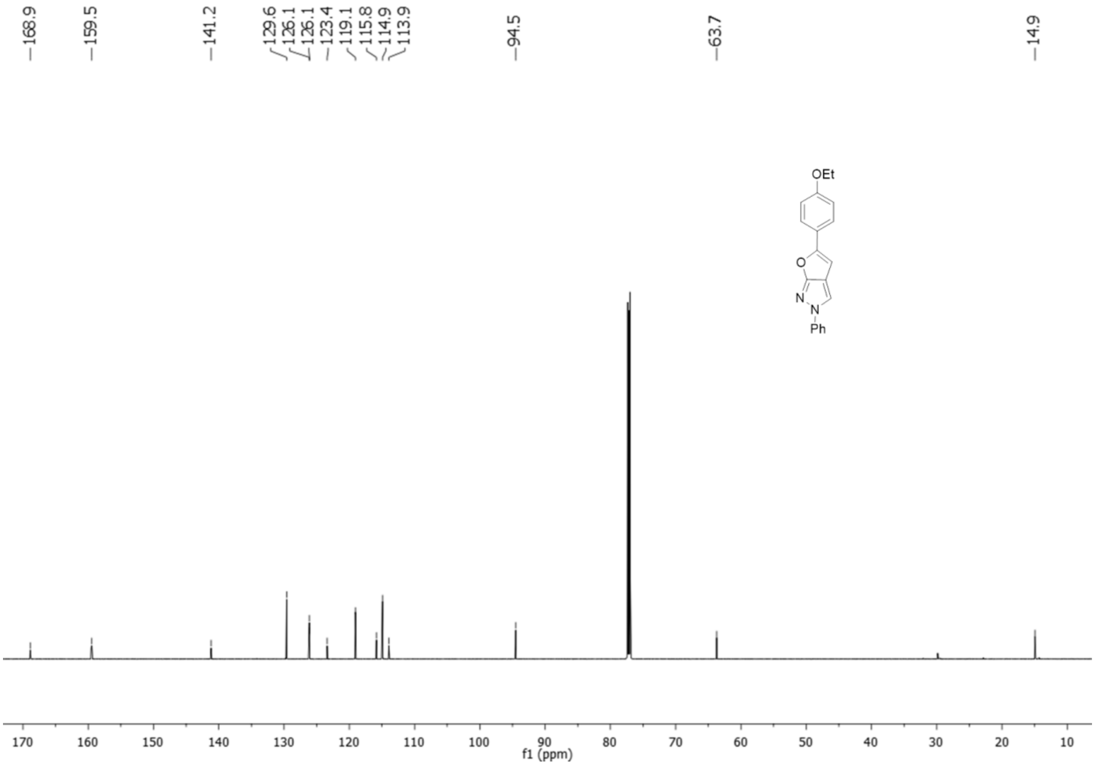

^{1}H NMR spectrum of 4f ($CDCl_3$, 700 MHz):

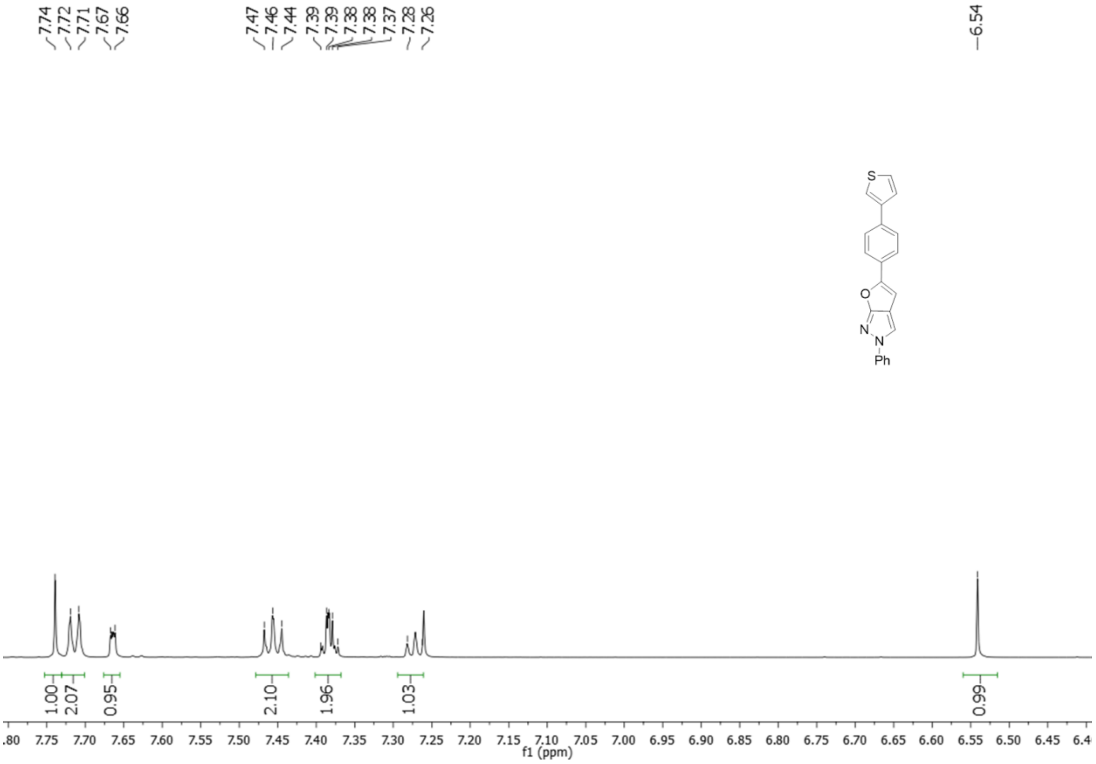

^{13}C NMR spectrum of 4f ($CDCl_3$, 176 MHz):

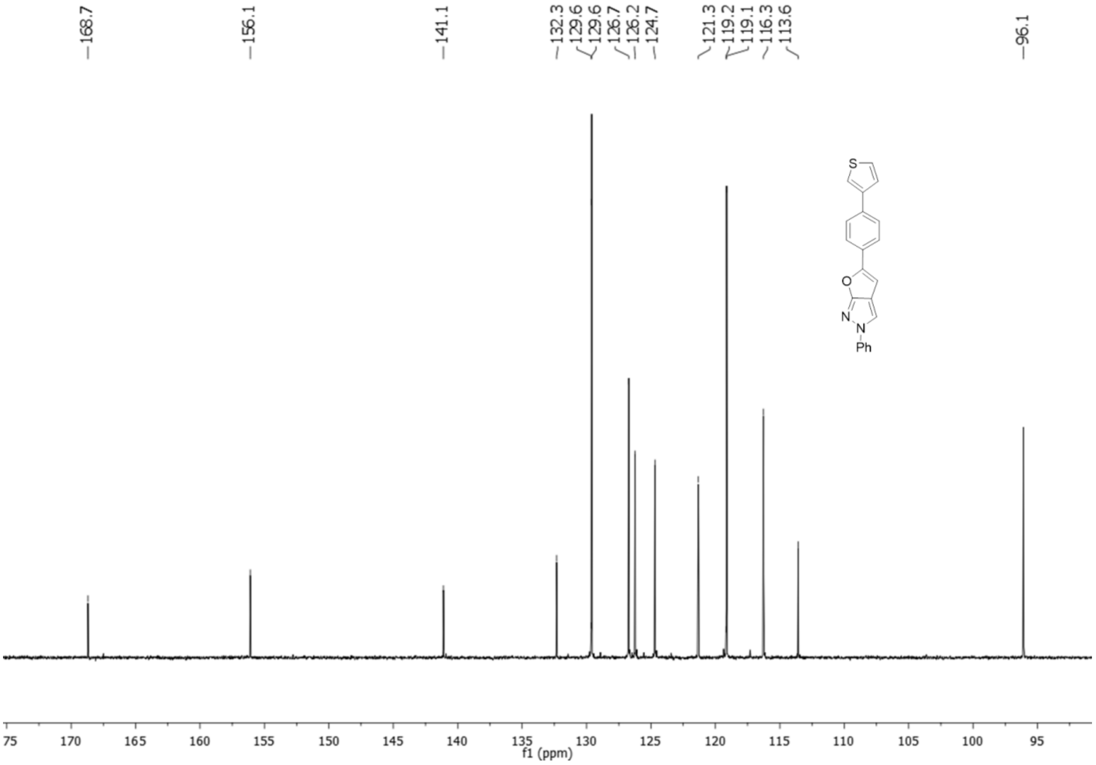

^{1}H NMR spectrum of 4g ($CDCl_3$, 700 MHz):

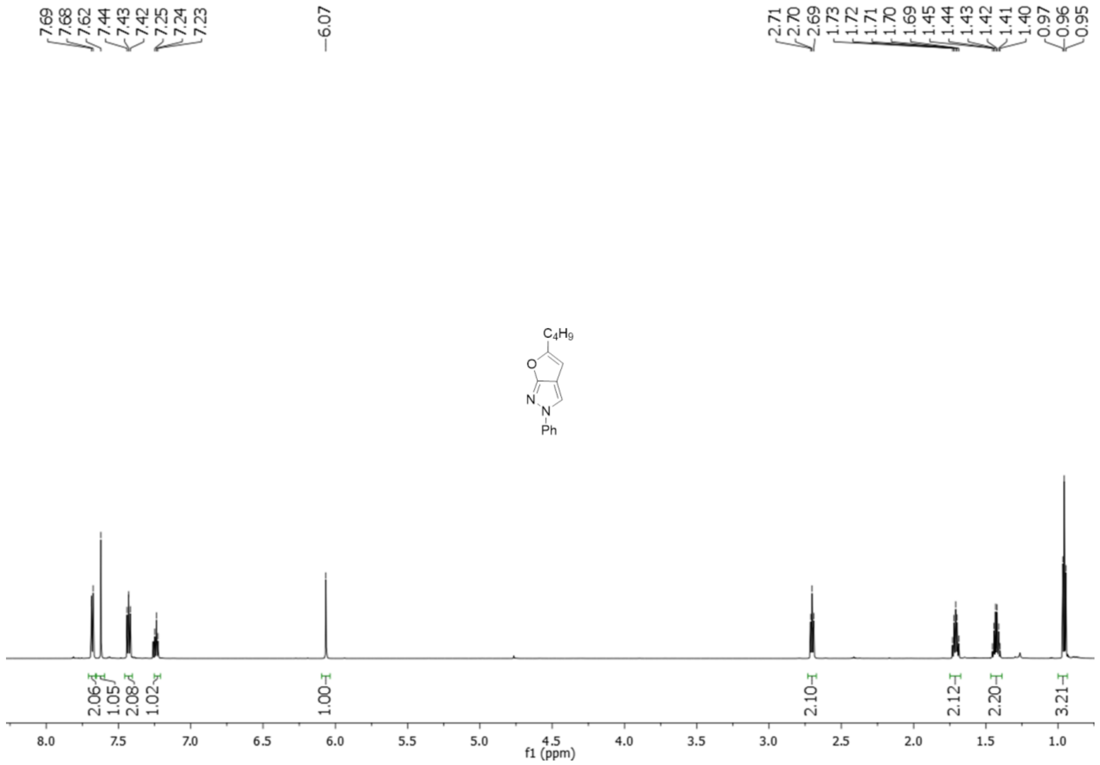

^{13}C NMR spectrum of 4g ($CDCl_3$, 176 MHz):

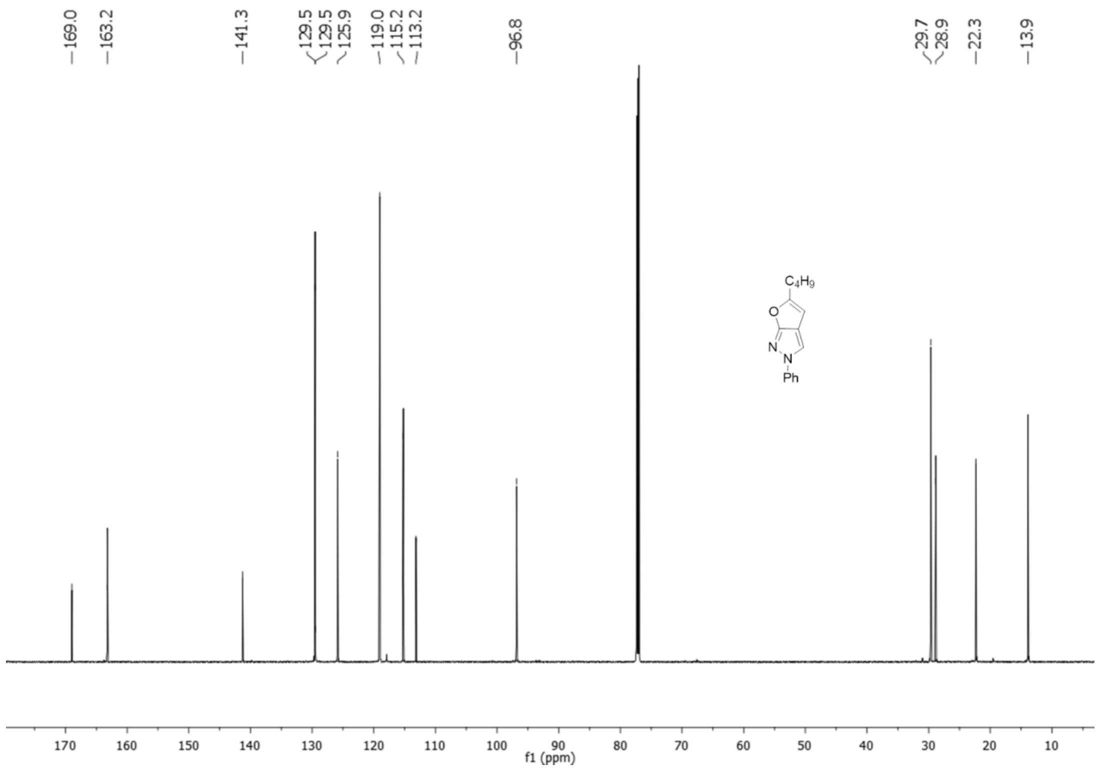

^{1}H NMR spectrum of 4h ($CDCl_3$, 700 MHz):

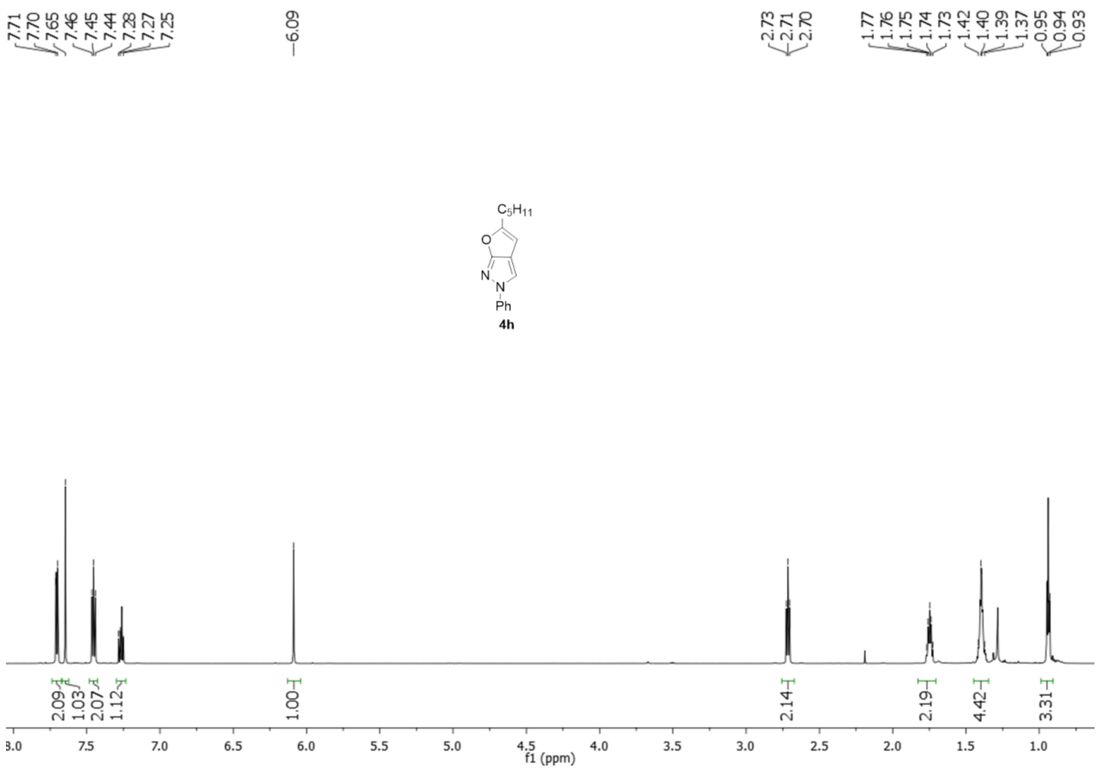

^{13}C NMR spectrum of 4h ($CDCl_3$, 176 MHz):

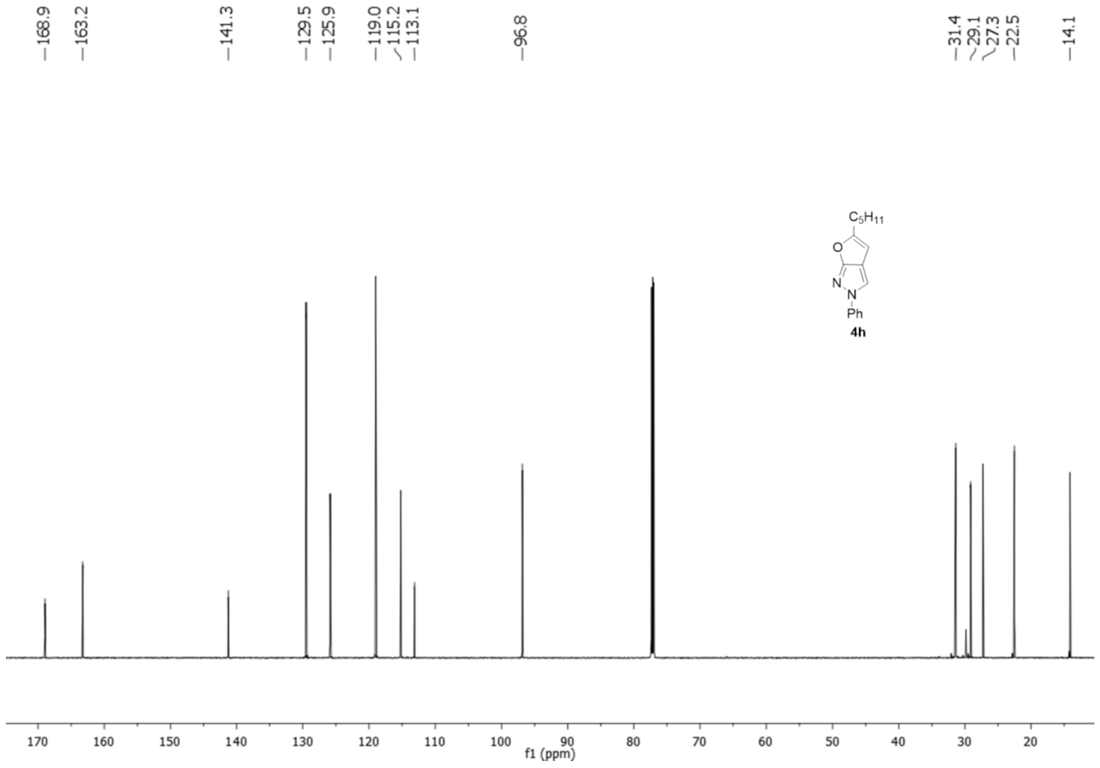

^{1}H NMR spectrum of 4j ($CDCl_3$, 700 MHz):

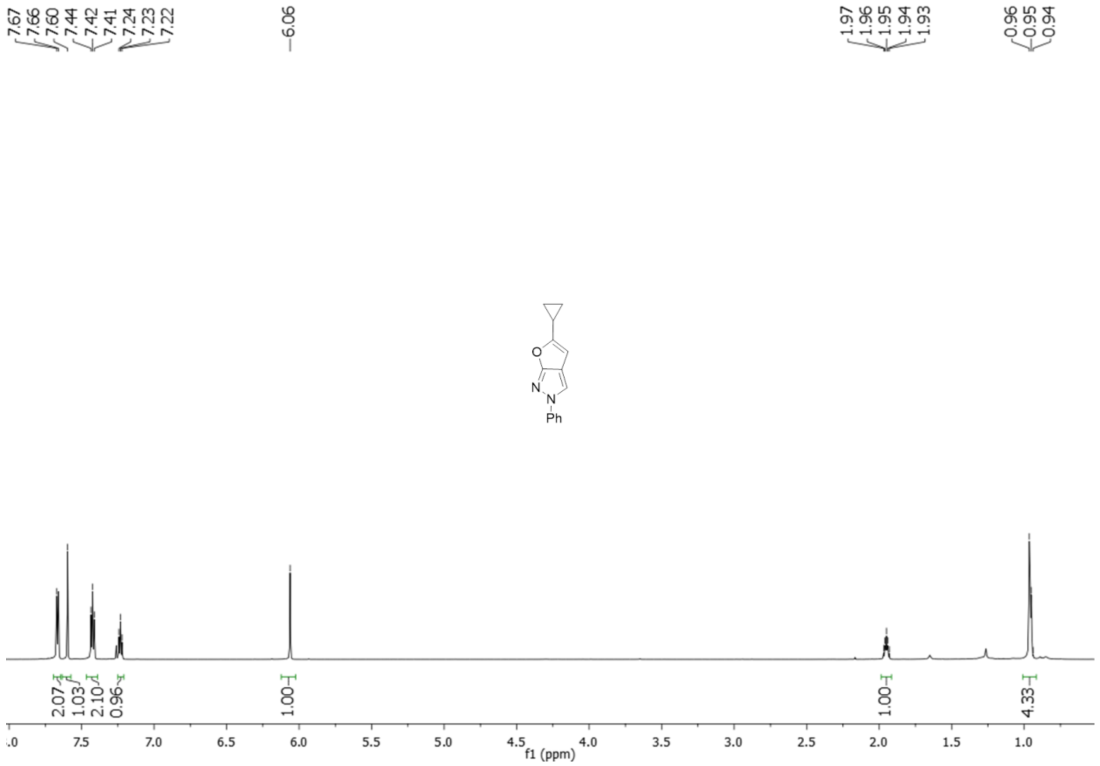

^{13}C NMR spectrum of 4j ($CDCl_3$, 176 MHz):

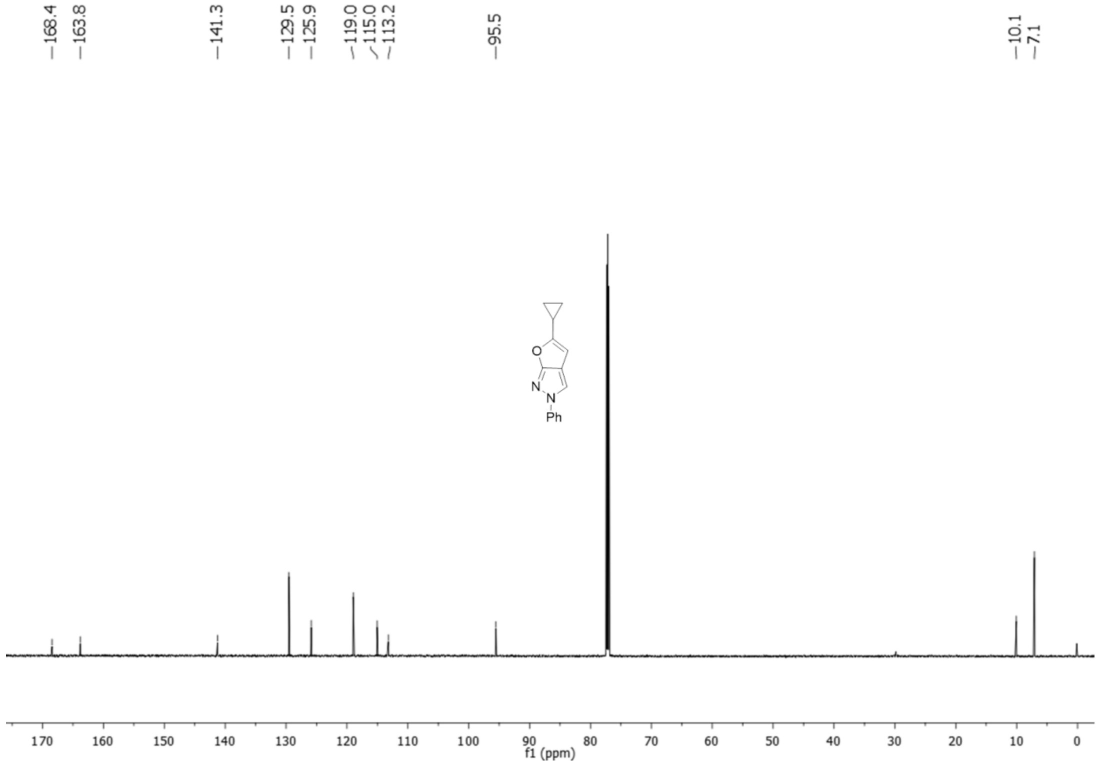

Supplement: File 1 — Experimental details and characterization data. [file Beilstein_J_Org_Chem-15-679-s001.pdf]
